# Acid tolerance and metabolic potential of comammox and nitrite-oxidizing *Nitrospira* enriched from soil

**DOI:** 10.1093/ismeco/ycaf167

**Published:** 2025-09-23

**Authors:** Yu Takahashi, Hirotsugu Fujitani, Itsuki Taniguchi, Yasuhiro Gotoh, Yuta Shimada, Shuto Ikeda, Tetsuya Hayashi, Kanako Tago, Masahito Hayatsu, Satoshi Tsuneda

**Affiliations:** Department of Life Science and Medical Bioscience, School of Advanced Science and Engineering, Waseda University, Shinjuku-ku, Tokyo, 162-8480, Japan; Department of Biological Sciences, Faculty of Science and Engineering, Chuo University, Bunkyo-ku, Tokyo, 112-8551, Japan; Department of Applied Physics and Chemical Engineering, Faculty of Engineering, Tokyo University of Agriculture and Technology, Koganei-shi, Tokyo, 184-8588, Japan; Department of Bacteriology, Graduate School of Medical Sciences, Kyushu University, Fukuoka-shi, Fukuoka, 812-8582, Japan; Advanced Genomics Center, National Institute of Genetics, Mishima-shi, Shizuoka, 411-8540, Japan; Department of Life Science and Medical Bioscience, School of Advanced Science and Engineering, Waseda University, Shinjuku-ku, Tokyo, 162-8480, Japan; Department of Life Science and Medical Bioscience, School of Advanced Science and Engineering, Waseda University, Shinjuku-ku, Tokyo, 162-8480, Japan; Department of Bacteriology, Graduate School of Medical Sciences, Kyushu University, Fukuoka-shi, Fukuoka, 812-8582, Japan; Department of Green Innovation and Environmental Bioscience, School of Veterinary Medicine, Kitasato University, Sagamihara-shi, Kanagawa, 252-0329, Japan; Institute for Agro-Environmental Sciences, National Agriculture and Food Research Organization (NARO), Tsukuba-shi, Ibaraki, 305-8604, Japan; Institute for Agro-Environmental Sciences, National Agriculture and Food Research Organization (NARO), Tsukuba-shi, Ibaraki, 305-8604, Japan; Department of Life Science and Medical Bioscience, School of Advanced Science and Engineering, Waseda University, Shinjuku-ku, Tokyo, 162-8480, Japan

**Keywords:** nitrification, acidic soil, acid tolerance, *Nitrospira*, comammox, complete ammonia oxidation, nitrite oxidation, metagenomics, enrichment culture

## Abstract

Nitrification is the two-step microbial oxidation of ammonia to nitrate via nitrite, and it can contribute to environmental problems in soils. Some nitrifiers have been cultivated from acidic soils at pH <5.5, allowing their metabolic potential and phylogeny to be investigated through genomic analyses. However, the genomic features of the genus *Nitrospira* remain poorly understood in the context of acid tolerance, despite its wide distribution in acidic environments. This study aimed to characterize the physiology and genomics of acid-tolerant *Nitrospira* enriched from an acidic soil. Using a metagenomic approach, two closed genomes of *Nitrospira* were reconstructed: a complete ammonia-oxidizing (comammox) bacterium and a nitrite-oxidizing bacterium (NOB). Both enriched *Nitrospira* survived at pH <5.5 in physiological tests, and the enriched comammox *Nitrospira* was phylogenetically close to clones derived from acidic soils. The active-site residues of hydroxylamine oxidase, a key nitrification enzyme, were conserved between the comammox *Nitrospira* characterized in this study and the previously reported betaproteobacterial ammonia oxidizers. This conservation suggests that existing nitrification inhibitors targeting this enzyme may also inhibit ammonia oxidation by comammox *Nitrospira* in acidic soils. Although the comammox and NOB *Nitrospira* in this study shared nearly all key metabolic pathways with *Nitrospira* species identified from neutral pH environments, both possessed passive urea transporters homologous to those found in acid-tolerant bacteria. These results revealed the acid tolerance of the enriched *Nitrospira* at pH <5.5, as well as their genomic features shared with acid-tolerant bacteria, rather than with previously reported *Nitrospira* species.

## Graphical Abstract

**Graphical Abstract:**
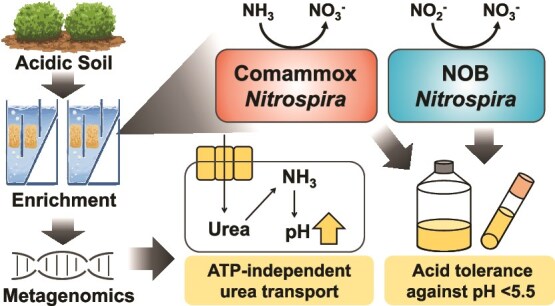

## Introduction

Nitrification is the sequential oxidation of ammonia to nitrate via nitrite and occurs in soils with a wide pH range, including acidic soils (pH <5.5) [1], which constitute most of the world’s potentially arable land [2]. In agricultural soils, nitrification converts ammonium from nitrogen fertilizers into nitrate, resulting in nitrogen loss [3, 4]. Furthermore, soil nitrification contributes to groundwater contamination with nitrate, soil acidification, and emissions of nitrous oxide, a potent greenhouse gas [5]. Therefore, the acid tolerance potential and underlying mechanisms in nitrifiers need to be understood.

Autotrophic microbial nitrification is carried out by ammonia-oxidizing bacteria (AOB), ammonia-oxidizing archaea (AOA), nitrite-oxidizing bacteria (NOB), and complete ammonia-oxidizing (comammox) bacteria. Conventionally, the growth and nitrification activity of nitrifiers are known to decrease markedly at pH <5.5 based on pure culture experiments [6]. However, novel acid-tolerant or acidophilic AOA and AOB have been enriched and/or isolated from acidic environments [7–10]. Furthermore, their metabolic potential and phylogeny have been elucidated by genomic approaches [9–12]. These studies have supported the concept that ammonia is oxidized to nitrite by AOA and AOB in acidic environments with a pH <5.5.

At pH <3.3, nitrite is chemically converted into nitrate and nitric oxide without biological mediation by nitrifiers [13]. However, under moderately acidic conditions (pH 3.3–5.5), nitrite can be oxidized by NOB or comammox bacteria. In fact, some acid-tolerant or acidophilic NOB have been reported. *Nitrobacter* sp. IOacid [14], *Candidatus* Nitrotoga sp. CP45 [15], and a *Nitrobacter* enrichment culture [16] survived at pH 4.1, 5.0, and 4.6, respectively. *Nitrobacter* sp. A67 [17] and *Ca.* Nitrobacter acidophilus [18] oxidized nitrite at pH 5.3 and 4.5, respectively.

Moreover, acid-tolerant *Nitrospira*, including NOB and comammox bacteria, have been widely detected in acidic environments such as biofilm reactors [6, 19], a membrane bioreactor [20], horticultural soilless media [21], a urine-fed bioreactor [22], a mine lake [23], and soils [24]. *Nitrospira* has been found in various acidic soils, including savanna soils [25], forest soils [26, 27], grassland soils [27], citrus orchard soils [28], tea field soils [29–31], paddy soils [32, 33], and some other agricultural soils [31, 34, 35]. Although these reports have supported the contribution of *Nitrospira* to nitrification in acidic soils mainly based on PCR-based approaches (e.g. quantitative PCR and amplicon sequencing), our previous study [36] demonstrated the enrichment of *Nitrospira* from acidic soils in laboratory bioreactors.

**Figure 1:**
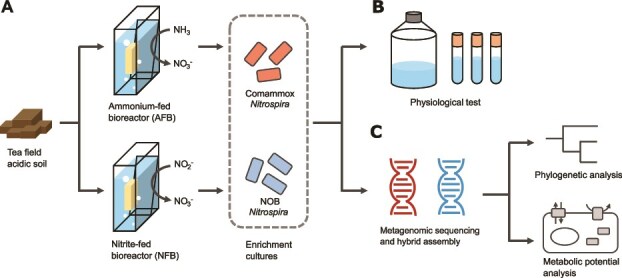
Experimental workflow of this study. (A) In our previous study, microbial biomass obtained from an acidic tea field soil was inoculated into AFB and NFB, resulting in the enrichment of comammox and NOB *Nitrospira* in the respective bioreactors. (B) Acid tolerance and recovery from acid exposure of the enrichments were evaluated through physiological tests. (C) Using metagenomic sequencing and hybrid assembly, genomes of the enriched *Nitrospira* were reconstructed, and their phylogeny and metabolic potential were analyzed.

Because culture-based studies on acid-tolerant *Nitrospira* from soils are still limited, their genomic features remain poorly understood, unlike those of acid-tolerant or acidophilic AOA and AOB. Hence, this study reports the genomic characteristics of acid-tolerant NOB and comammox *Nitrospira* enriched in bioreactors (Fig. 1). In our previous study [36], two bioreactors, ammonium-fed bioreactor (AFB) and nitrite-fed bioreactor (NFB), were operated at pH 5.5, and *Nitrospira* lineage II accounted for >50% of all bacteria in both systems, based on 16S ribosomal RNA (rRNA) gene analysis [36]. By analyzing these enrichment cultures using physiological tests and a metagenomic approach, this study aimed to investigate acid-tolerant *Nitrospira* in terms of (i) tolerance against pH <5.5, (ii) phylogenetic novelty, and (iii) genetic potential for nitrogen metabolism and acid tolerance.

## Materials and methods

### Operation of bioreactors

The details of the bioreactor setup and operational procedures are described in our previous study [36]. In brief, microbial biomass obtained from an acidic tea field soil was transferred into two bioreactors, AFB and NFB, which were supplied with inorganic media containing NH_4_Cl and NaNO_2_, respectively. These bioreactors were equipped with a continuous feeding system [37] that had previously been used to enrich comammox *Nitrospira* [38]. The substrate concentrations in the media were gradually increased from 0.07 to 50 mM for NH_4_Cl, and from 0.07 to 0.36 mM for NaNO_2_. The pH of the media in both bioreactors was initially adjusted to 6.0 until nitrification activity stabilized, after which it was lowered to 5.5 to enrich acid-tolerant nitrifiers.

### Acid tolerance evaluation of AFB *Nitrospira* enrichment

The detailed procedures are described in Supplementary Information (SI). In brief, the following procedures were conducted in biological duplicate using two bottles, designated Bottle 1 and Bottle 2. Enrichment culture was collected from AFB. Centrifugation, supernatant removal, medium addition, and dispersion were performed twice to remove residual supernatant. The bacterial cells were then resuspended in fresh inorganic medium (Table S1) adjusted to pH 6 and supplemented with 5 mM NH_4_Cl. Bottles containing AFB enrichment were incubated statically in the dark at room temperature. The pH of the medium (pH_medium_) was measured every few days, and 500 μl of a 50 g/l NaHCO_3_ solution was added on Days 7, 13, and 25 to increase the pH_medium_ to 4.28–5.92. Culture samples were collected on Days 0, 7, 10, 13, 20, 25, 26, and 28 to measure ammonia, nitrite, and nitrate concentrations.

After 28 days of incubation, 160 ml of culture was collected from Bottle 2. The bacterial cells were harvested by centrifugation and resuspended in 300 ml of fresh medium. The suspension was transferred to a new bottle, designated Bottle 3, and incubated using the same method as for Bottle 2.

### Test for recovery from acidic stress of NFB *Nitrospira* enrichment

The detailed procedures are described in SI. In brief, enrichment culture was collected from NFB. Centrifugation, supernatant removal, medium addition, and dispersion were performed in the same manner as for AFB samples. The washed bacterial cells were suspended in medium (Table S2) adjusted to pH 3.3 or 2.4 and buffered with 2-morpholinoethanesulfonic acid (MES). Cells from NFB were exposed to pH 3.3 or 2.4 statically for 7 days. During the acid exposure period, the pH_medium_ was measured daily. After exposure, centrifugation, supernatant removal, medium addition, and dispersion were repeated twice to wash out the MES-containing supernatant. The washed bacterial cells were then resuspended in fresh medium at pH 8 and transferred into three glass test tubes (in biological triplicate). NaNO_2_ was added to the suspensions at a final concentration of 720 μM, and the tubes were incubated statically in the dark at room temperature. Culture samples were collected to measure nitrite concentrations, which were then used to calculate nitrite consumption rates.

### Test for spontaneous degradation of nitrite

The medium for NFB was prepared using the same method as described above. It was buffered with 80 mM MES, and the initial pH_medium_ was adjusted to pH 2.7 or 5.6. Nitrite was added to a final concentration of 714 μM, and 30 ml of the medium was transferred into glass tubes. After 2 days of static incubation in the dark at room temperature, the nitrite concentration in the medium was measured.

### Test for nitrification rates of AFB enrichment at different ammonia concentrations

The detailed procedures are described in SI. In brief, enrichment culture was collected from AFB, and the residual supernatant was removed using the same method as described above. The washed bacterial cells were incubated for 5 days at different initial NH_4_Cl concentrations (0, 0.5, 1.0, 2.5, 5.0, or 10 mM). Culture samples were collected on Days 0, 1, 2, and 5 to measure pH_medium_ and nitrate concentrations.

### Test for nitrification rates of NFB enrichment at different nitrite concentrations

The detailed procedures are described in SI. In brief, enrichment culture was collected from NFB, and the residual supernatant was removed as described above. The washed cells were suspended in medium adjusted to pH 7.2 and supplemented with 0, 0.71, 3.6, 7.1, 21, 36, or 71 mM NaNO_2_ and incubated for 4 days. Culture samples were collected daily for the measurement of nitrate concentrations.

### Measurement of total ammonia, nitrite, and nitrate concentrations

The concentration of total ammonia, defined as the sum of free ammonia (NH_3_) and ammonium ion (NH_4_^+^), was determined using the indophenol ion reaction [39], with absorbance measured at 630 nm. Due to the large measurement error associated with this method, measurements were performed in technical triplicate. Nitrite concentration was determined by the Griess–Romijn reagent [40], with absorbance measured at 560 nm. Nitrate was first reduced to nitrite using vanadium(III) chloride, and its concentration was then determined using the Griess–Romijn reagent, with absorbance measured at 540 nm, following a previously reported method [40]. All absorbance measurements were conducted using a microplate reader Synergy H1 (Agilent, Santa Clara, CA, USA). Exceptionally, for the experiments “Test for spontaneous degradation of nitrite” and “Test for nitrification rates of NFB enrichment at different nitrite concentrations,” nitrite and nitrate concentrations were measured using an IC-2010 ion chromatography system equipped with a TSKgel SuperIC-Anion HS (Tosoh Corporation, Tokyo, Japan).

### Microscopic observation

On Day 26 of the acid tolerance evaluation of AFB enrichment, 500 μl of culture was collected. The same volume was collected from NFB enrichment after 7 days of exposure to pH 2.4. The samples were dispersed using a Q55 sonicator (Qsonica LLC, Newtown, PA, USA) with intermittent sonication for 20 s at 20% amplitude. Fluorescence in situ hybridization (FISH) was performed according to the standard protocol [41]. The oligonucleotide probe S-^*^-Ntspa-1151-a-A-20 [42], fluorescently labeled with a hydrophilic sulfoindocyanine dye (Cy3), was used to stain *Nitrospira* lineage II cells. The SYTOX Green nucleic acid stain (Thermo Fisher Scientific, Waltham, MA, USA) was used to stain microbial nucleic acids in the cultures. Stained cells were observed using a microscope BX51 (Olympus, Tokyo, Japan).

To evaluate cell dispersion by prolonged sonication, enrichment culture freshly collected from NFB was sonicated for 0, 30, 60, 90, and 120 s. Sonication, FISH, nucleic acid staining, and microscopic observation were performed using the same methods as described above.

### Genomic DNA extraction

The detailed procedures, materials, and reagents are described in SI. Briefly, bacterial cells were collected from AFB and NFB by centrifugation and lysed using a combination of physical, chemical, and enzymatic methods, including bead beating, multiple lysis reagents, and proteinase K treatment. Genomic DNA in the lysates was purified using column filters and wash buffers.

### Metagenomic sequencing and hybrid assembly

The detailed procedures, materials and software are described in SI. Briefly, DNA libraries were prepared and sequenced using HiSeq (Illumina, San Diego, CA, USA) and MinION (Oxford Nanopore Technologies). The raw reads were trimmed and used for hybrid assembly and binning. To minimize undesirable noises arising from the use of excessive read numbers in assembly, various combinations of read subsets were tested, and those that yielded the highest-quality *Nitrospira* metagenome-assembled genomes (MAGs) were selected (Table S3). According to a previous report [43], MAGs with high completeness (≥90%) and low contamination (≤10%) were considered high-quality MAGs and were used for subsequent analyses. The relative abundances of MAGs were estimated by mapping the HiSeq reads.

### Functional gene annotation

Genes in the reconstructed MAGs were functionally annotated using DFAST version 1.2.16 [44] with default settings. For annotation of nitrifier MAGs, reference genomes were obtained from the National Center for Biotechnology Information (NCBI) Assembly database [45] (Table S4). The remaining MAGs were annotated without specifying reference genomes.

### Phylogeny and identity analyses of *Nitrospira* MAGs

To identify *Nitrospira* genomes among the high-quality MAGs, sequences highly homologous to their 16S rRNA genes were searched for in the NCBI Nucleotide database using NCBI BLASTn [46]. For MAGs lacking 16S rRNA genes, the RpoB or RpoA amino acid sequences were used as queries to search for homologs, as the *rpoB* and *rpoA* genes are used as taxonomic markers [47, 48].

For phylogenetic analysis, whole genome sequences of *Nitrospira* and *Leptospirillum* were obtained from the NCBI Assembly database [45] (Table S5). Using default settings, sequences of 91 single-copy marker genes were extracted from the assemblies and aligned based on codons using the up-to-date bacterial core gene (UBCG) pipeline [48]. A phylogenetic tree was constructed using the maximum likelihood (ML) method based on the Tamura–Nei model [49] in MEGA11 [50], and bootstrap values were calculated from 100 iterations.

To identify the genomes closely related to the reconstructed *Nitrospira* MAGs in this study, 456 genomes (all assemblies registered as *Nitrospira*; accessed on 19 April 2022) were downloaded from the NCBI Assembly database. For each of AFB01 and NFB02, Mash distances [51] were calculated against all assemblies. The five genomes with the lowest Mash distances were selected, and their average nucleotide identity (ANI) and average amino acid identity (AAI) relative to AFB01 and NFB02 were calculated. ANI was calculated using pyani [52] with the BLAST+ alignment algorithm, and AAI was calculated using EzAAI [53] with default settings.

### Metabolic pathway annotation

To identify proteins involved in energy and carbon metabolism, all amino acid sequences of AFB01 and NFB02 were analyzed using the Kyoto Encyclopedia of Genes and Genomes (KEGG) internal tool GhostKOALA [54]. BLAST searches were conducted against the “genus_prokaryotes” database, and proteins assigned K numbers corresponding to KEGG modules for “energy and carbon metabolism” were listed.

Proteins involved in nitrogen metabolism (including nitrification, nitrogen assimilation, urea degradation, and urea transport), superoxide detoxification, formate metabolism, hydrogen metabolism, and multiple resistance and pH adaptation (Mrp) cation/proton antiporters were identified using the NCBI BLASTp [46] with default parameters. Previously annotated proteins involved in these pathways were used as queries, and all amino acid sequences of AFB01 and NFB02 were used as subjects.

Homologs of acid tolerance–related proteins were identified using the BLASTp tool from BLAST+ version 2.16.0 [55]. Known acid tolerance–related proteins were used as queries, and all amino acid sequences of AFB01 and NFB02 were used as subjects. Subject sequences with an e-value <1 × 10^−5^ were considered homologs of the query sequences. In addition, all proteins of *Nitrospira japonica* and *Candidatus* Nitrospira kreftii (accession numbers: GCA_900169565.1 and GCA_014058405.1, respectively), retrieved from the NCBI Assembly database [45], were analyzed using the same method and served as references.

### Phylogenetic analysis of selected genes

To analyze the phylogeny of *Nitrospira* MAGs in this study, reference sequences for the ammonia monooxygenase subunit A (*amoA*) gene (24 nucleotide sequences), hydroxylamine oxidoreductase (HAO) (20 amino acid sequences), and urea transporter (UT) (25 amino acid sequences) (Table S6) were retrieved from the NCBI databases [56]. For *amoA*, sequences from cultured comammox *Nitrospira* (clades A and B), *Nitrosomonas* (outgroup), and clones derived from acidic soils were selected. For HAO, sequences from comammox *Nitrospira*, AOB (*Betaproteobacteria* and *Gammaproteobacteria*), and methane oxidizers (outgroup) were used. For UT, sequences from *Nitrospira*, *Desulfovibrionaceae* (a well-known lineage in studies on UT), *Pseudomonadota* (AOB and acid-tolerant bacteria), and AOA (outgroup) were selected.

Alignment and ML phylogenetic analyses were performed using MEGA11 [50]. The Tamura–Nei model [49] and the Jones–Taylor–Thornton model [57] were applied to nucleotide and amino acid sequences, respectively. Bootstrap values were calculated based on 500 iterations.

### Quantification of AFB01-related sequences in soils

The detailed procedures are described in SI. Briefly, previously reported comammox *amoA* amplicon sequences [29] were analyzed to estimate the relative abundance of AFB01-related sequences within comammox *Nitrospira* community in soils. Following a previous study [58], raw reads were processed using DADA2 [59] to obtain amplicon sequence variants (ASVs), and AFB01-related ASVs were identified using BLAST+ [55].

### Alignment of HAO amino acid sequences

The HAO amino acid sequences of AFB01 and homologs from the database were aligned using homology-extended multiple alignments using the PRALINE toolbox [60]. The putative active sites of AFB01 HAO were estimated based on the active-site residues of *Nitrosomonas europaea* HAO, which are arranged around the pocket on the catalytic heme P460 [61].

### Comparative genomics

The detailed procedures are described in SI. Briefly, the amino acid sequences of all proteins from AFB01 and NFB02 were compared with those of representative *Nitrospira* spp. using OrthoVenn2 [62], and proteins unique to AFB01 and NFB02 were identified.

### Genomic context visualization of the UT genes

The genomic contexts surrounding the UT genes of AFB01 and NFB02 were visualized using Gcluster [63], with reference to the gbff files of *N. japonica* and *N. inopinata* (accession numbers: GCA_900169565.1 and GCA_001458695.1, respectively), which were retrieved from the NCBI Assembly database [45].

## Results and discussion

### Acid tolerance and recovery from acid exposure of nitrifier enrichments

In our previous study [36], *Nitrospira* lineage II was more enriched than other AOB and NOB. The acid tolerance and recovery from acid exposure of these enrichments were evaluated through physiological tests.

**Figure 2:**
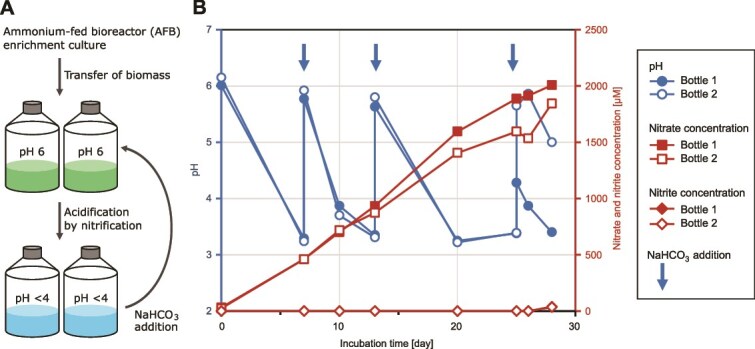
Ammonia oxidation by AFB enrichment under acidic conditions. (A) Schematic illustration of the cultivation process. (B) pH (circles), nitrate concentrations (squares), and nitrite concentrations (diamonds) in the medium. Filled and open symbols represent two biological replicates. Vertical arrows indicate the timing of NaHCO_3_ supplementation at 7, 13, and 25 days of incubation.

First, the nitrification activity of AFB enrichment at pH <5.5 was confirmed. The enrichment was suspended in an inorganic medium containing 5 mM NH_4_Cl and incubated statically. Because ammonia oxidation is an acid-producing reaction [6], NaHCO_3_ solution was supplemented to increase the pH_medium_ above 4 (Fig. 2A). The pH_medium_ decreased from 6.1 ± 0.1 to 3.3 ± 0.04 after 7 days of incubation (Fig. 2B). The pH_medium_ was increased by adding NaHCO_3_ solution on Day 7 but decreased again to pH 3.3 ± 0.03 on Day 13. Acidification and NaHCO_3_ supplementation were repeated, and the pH_medium_ fluctuated between 3.2 and 6.1 (Fig. 2B). During this period, the nitrate concentration increased to 1.9 ± 0.1 mM by Day 28, whereas the nitrite concentration remained below 0.0025 mM (Fig. 2B). A total of 1.8–2.2 mM of ammonia was consumed during this time, which was approximately consistent with the combined concentrations of the produced nitrite and nitrate (Fig. S1). Furthermore, *Nitrospira* lineage II cells were detected by FISH after 26 days of incubation (Fig. S2A and B), demonstrating that *Nitrospira* cells remained detectable after exposure to pH <5.5. In addition, after 28 days of incubation, the bacterial cells in the enrichment culture were transferred to fresh medium and exhibited nitrification activity (Fig. S3), indicating that the enrichment retained its nitrification activity after exposure to pH <5.5.

**Figure 3:**
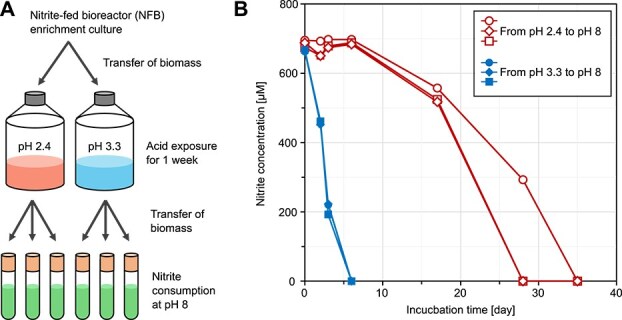
Nitrite consumption by NFB enrichment after acid exposure. (A) Schematic illustration of the acid exposure and subsequent nitrite consumption under neutral conditions. (B) Nitrite concentrations of three biological replicates (represented by circle, square, and diamond symbols) at pH 8 following acid exposure (open symbols, pH 2.4; filled symbols, pH 3.3).

Next, recovery from temporary acid exposure was tested using NFB *Nitrospira* enrichment culture. In our previous study [36], the nitrification activity of NFB enrichment was inhibited by free nitrous acid (FNA) rather than by acidity. Moreover, nitrite spontaneously degraded in buffered medium at pH 2.7–5.6 (Fig. S4), making it difficult to assess nitrite oxidation activity under acidic conditions. Therefore, a nitrite-free medium was used in this experiment to specifically evaluate the effects of acidity, excluding the influence of FNA toxicity and spontaneous nitrite degradation. In the first stage of the experiment, NFB enrichment was exposed to acidic conditions (a moderately acidic condition at pH 3.3 and a more strongly acidic condition at pH 2.4) for one week in a nitrite-free medium. In the second stage, nitrite consumption was tested at pH 8 (Fig. 3A), as neutral to slightly alkaline conditions are optimal for the growth of NOB [6].

NFB enrichment was suspended in two bottles containing nitrite-free medium. The pH_medium_ was maintained at pH 2.2–2.6 (2.4 ± 0.2) and 3.1–3.6 (3.3 ± 0.2), respectively (Fig. S5). After 1 week of acid exposure, the cells were collected and transferred to test tubes to evaluate nitrite consumption at pH 8 (Fig. 3A). Cells transferred from pH 3.3 to pH 8 consumed nitrite within six days (Fig. 3B), whereas those transferred from pH 2.4 did not start nitrite consumption until after Day 6, but completely consumed the nitrite within 35 days (Fig. 3B). Accordingly, the mean nitrite consumption rates of the cells exposed to pH 3.3 were higher than those exposed to pH 2.4 (Fig. S6). However, the rates for biomass exposed to pH 2.4 may have been underestimated, as they were not normalized to the nitrifier biomass. Notably, the nitrite consumption activity of NFB enrichment was not entirely lost after exposure to pH 2.4 (Fig. 3B), and *Nitrospira* lineage II cells labeled by FISH were detected in cultures exposed to pH 2.4 for 7 days (Fig. S2C and D).

These results demonstrate that the tested enrichments, including comammox and NOB *Nitrospira*, were not completely inactivated after exposure to pH <5.5. However, it should be noted that AFB enrichment contained nitrifiers other than *Nitrospira*, such as *Nitrosospira* and *Nitrobacter*, and that NFB enrichment contained at least two phylotypes of *Nitrospira* [36]. Moreover, the community structures of the nitrifiers were not assessed during incubation, and viable cell counts of *Nitrospira* by serial dilution could not be determined because their aggregates were difficult to disperse into single cells by sonication (Fig. S7). Additionally, short-term incubations were technically challenging, which hindered attempts to minimize changes in community structure during the incubations (see Figs S8 and S9 and SI for the detailed results and discussion). Therefore, it cannot be ruled out that the relative abundances and/or viable cell numbers of the enriched *Nitrospira* in AFB and NFB decreased due to acid exposure and that several species of nitrifiers contributed to nitrification.

### Phylogenomics

Using metagenomic sequencing and hybrid assembly, 16 and 8 high-quality MAGs were reconstructed from AFB and NFB enrichments, respectively (Tables S7–S9). Among them, one MAG from AFB (AFB01) and two MAGs from NFB (NFB01 and NFB02) were identified as relatives of known *Nitrospira* (Table S10), and their genomic features were characterized (Table S11). AFB01 and NFB02 were closed (complete) genomes (Table S8) with 0% strain heterogeneity (Table S7). In contrast, NFB01 consisted of 22 contigs (Table S8) and showed a strain heterogeneity of 71.43% (Table S7), indicating that NFB02 was of higher quality than NFB01.

In addition to *Nitrospira*, two other nitrifier MAGs were reconstructed, *Nitrobacter* sp. AFB05 and *Nitrosospira* sp. AFB09 (Table S10), suggesting that multiple nitrifiers coexisted in these bioreactors. However, relative abundance analysis revealed that AFB01 and NFB02 were more abundant than other MAGs in AFB and NFB, respectively (Fig. S10), indicating that AFB01 and NFB02 were the predominant nitrifiers in their respective bioreactors.

**Figure 4:**
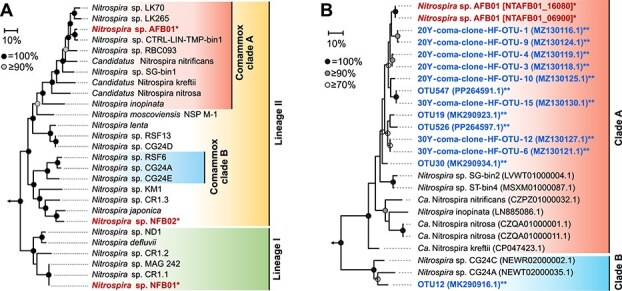
Phylogenetic analyses of *Nitrospira*. MAGs retrieved in this study are shown in bold font with single asterisks (^*^), and clones derived from acidic soils are shown in bold font with double asterisks (^**^). Shading indicates lineages and clades. Scale bars correspond to 10% estimated sequence divergence. (A) Phylogenetic tree based on 91 single-copy genes. Bootstrap support from 100 iterations is shown by circles. The outgroup, consisting of *Leptospirillum* species, is indicated by an arrow. (B) Phylogenetic tree based on nucleotide sequences of the comammox *amoA* gene. Bootstrap support from 500 iterations is indicated by circles. Letters in parentheses and square brackets indicate accession numbers and locus tags, respectively. The outgroup, consisting of *N. europaea* ATCC 19178 (JN099309.1) and *N. eutropha* Nm 57 (KU747123.1), is indicated by an arrow.

The phylogeny of the reconstructed *Nitrospira* MAGs was analyzed using single-copy marker genes. AFB01 and NFB02 were classified into lineage II, whereas NFB01 belonged to lineage I (Fig. 4A). *Nitrospira* lineage II includes NOB and comammox bacteria [64] and comammox *Nitrospira* branches into clades A and B [65]. Based on phylogenetic analyses, AFB01 and NFB02 were classified into comammox clade A and NOB, respectively (Fig. 4A). In our previous study, AFB and NFB were inoculated with an acidic tea field soil, and *Nitrospira* lineage II was enriched in both bioreactors [36]. Functional genes of *Nitrospira* lineage II and comammox clade A were detected in acidic tea field soils at pH 3.35 and 3.83 [29]. The phylogenetic positions of AFB01 and NFB02 were consistent with environmental clones and enriched lineages.

To assess the novelty of AFB01 and NFB02, Mash distances were calculated against publicly available *Nitrospira* assemblies. The five assemblies with the lowest Mash distances were then selected for ANI and AAI analyses. The results indicated that NFB02 was distantly related to any public genome. ANI and AAI values between NFB02 and its closest relative, *N. japonica*, were 84.0% and 87.4%, respectively (Table S12). In contrast, AFB01 was closely related to a previously reported assembly, CTRL-LIN-TMP-bin1 with ANI and AAI values of 94.8% and 95.0%, respectively (Table S12). Given that 95% is a commonly used threshold for species demarcation [66], the two MAGs may be classified as the same species. Unexpectedly, however, CTRL-LIN-TMP-bin1 was reconstructed from a bioreactor operated at pH ~7.4 and inoculated with activated sludge from a wastewater treatment plant [67]. This finding suggests that a close relative of AFB01 has already been discovered in a neutral pH environment, implying that AFB01 and its close relatives may be acid-tolerant rather than acidophilic and capable of surviving in both neutral and acidic environments.

To examine the relationship among AFB01, NFB02, and *Nitrospira* clones previously detected in a biofilm reactor operated at pH 4.3 [6], a phylogenetic tree based on the 16S rRNA gene was constructed. Although these clones were classified within lineage II, they were distantly related to AFB01 and NFB02 (Fig. S11), indicating that lineage II includes acid-tolerant *Nitrospira*, other than close relatives of AFB01 and NFB02. Furthermore, *Nitrospira* lineage II also contributes to nitrification in alkaline lakes [68], suggesting that this lineage may comprise various acid- and alkali-tolerant species.

Moreover, the phylogeny of AFB01 was analyzed based on *amoA* genes. AFB01 possessed two copies of *amoA* (Dataset S1), and their sequences were identical (Fig. 4B). AFB01 was more closely related to clones derived from acidic soils than to previously isolated or enriched comammox *Nitrospira*, such as *N. inopinata*, *Ca.* N. nitrosa, *Ca.* N. nitrificans, and *Ca.* N. kreftii. Close relatives of AFB01 have been detected in various acidic soils. 20Y- and 30Y- coma-clone-HF-OTUs were identified from ^13^C-labeled DNA in a stable isotope probing analysis of orchard soils at pH 4.96 and 4.29, respectively [28]. OTU19 and OTU30 were detected in agricultural soils at pH 5 [34]. OTU526 and OTU547 were identified in tea field soils at pH 3.68 [30]. Additionally, *amoA* ASVs closely related to AFB01 were detected in acidic tea field soils with pH 3.35–3.83 and were more abundant than those in soils with higher pH (Fig. S12).

However, not all comammox *Nitrospira* from acidic soils belonged to the AFB01-related group in clade A. For example, OTU12 detected in soils at pH 5 [34] belonged to clade B (Fig. 4B). The presence of comammox clade B in acidic soils has been reported in several studies [26, 35]. Thus, not only acidic pH but also other environmental factors may contribute to niche partitioning among comammox *Nitrospira* species inhabiting acidic soils. Previous studies on the abundance [69, 70] and community composition [70] of soil comammox *Nitrospira* have shown that the two clades respond differently to nitrogen fertilizer application. Other factors, such as temperature, aeration, and soil moisture, also influence nitrifier communities [4].

As described above, comammox *amoA* genes have been detected in various acidic soils using PCR-based approaches. To our knowledge, however, no comammox *Nitrospira* MAGs have been directly reconstructed from acidic soils with pH <5.5, likely due to the low abundance of *Nitrospira* in soil microbial communities. Indeed, 16S rRNA gene analyses revealed that *Nitrospira* accounted for only 0.1% and 1.2% of the bacterial community in acidic soils from savanna [25] and tea fields [36], respectively. To overcome this limitation, a culture-based approach combined with metagenomics was necessary. In this context, AFB enrichment culture enabled metagenomic analysis of AFB01 and physiological tests supporting its acid tolerance. This would help us understand the genomics and physiology of uncultured AFB01-related species in acidic soils.

Based on phylogenetic analysis of HAO amino acid sequences, comammox *Nitrospira*, including AFB01, were more closely related to betaproteobacterial than to gammaproteobacterial AOB (Fig. S13), consistent with previous studies [65, 71]. To further investigate the features of AFB01 HAO, its amino acid sequence was aligned with those of beta- and gamma-proteobacterial AOB. The crystal structure of HAO from *N. europaea*, a beta-AOB [72], along with its six residues in the substrate-binding pocket [61], has been reported. These six residues in AFB01 HAO were identical to those of beta-AOB species (*N. europaea* and *Nitrosospira multiformis*), but differed from those in *Nitrosococcus oceanii*, a gamma-AOB (Fig. S14). *N. inopinata*, a comammox bacterium, also conserved these six residues in its HAO (Fig. S14).

These results suggest that HAOs of comammox *Nitrospira*, including AFB01, conserve active-site residues found in beta-AOB HAOs. This is consistent with a previous clone library analysis, which showed that comammox *hao* genes, retrieved from acidic tea field soils, highly conserved these residues [29]. Although further experiments are required, the characteristics of AFB01 HAO support the hypothesis that HAO activity in both beta-AOB and comammox bacteria can be inhibited by a common nitrification inhibitor [29].

### Metabolic potential and genomic adaptations to acidic conditions

The metabolic potential of AFB01 and NFB02 was assessed through metabolic pathway analysis (Table S13). Overall, the key metabolic pathways in AFB01 and NFB02 were largely consistent with those found in previously characterized *Nitrospira* genomes. Likewise, well-known acid tolerance-related proteins were not unique to AFB01 and NFB02, as most of them were also conserved in other *Nitrospira* species (Table S14) (see SI for the detailed results and discussion). Moreover, most proteins identified in AFB01 and NFB02 but absent from other representative *Nitrospira* spp. (Datasets S2 and S3) were unrelated to acid tolerance.

Unexpectedly, both AFB01 and NFB02 possessed the Mrp cation/proton antiporter (Table S13). Mrp antiporters contribute to survival in saline and/or alkaline environments (see SI for a detailed discussion). The Mrp system has been identified in *Ca.* N. alkalitolerans, haloalkalitolerant *Nitrospira* enriched from saline-alkaline lakes [73]. However, the *mrp* genes have also been found in *Nitrospira* MAGs reconstructed from nonsaline-alkaline environments, such as terrestrial subsurface sediments [74] and an acid mine lake [23]. These findings suggest that although Mrp antiporters may support tolerance to saline and/or alkaline conditions, their presence is not restricted to *Nitrospira* from such habitats.

**Figure 5:**
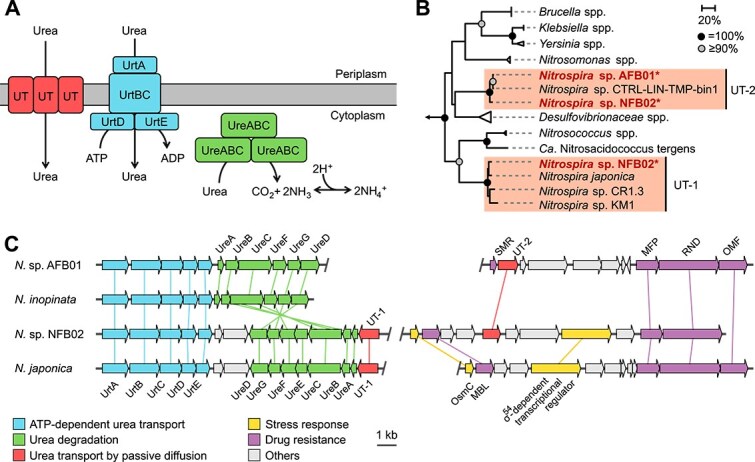
Genome-based characterization of urea transporters in acid-tolerant *Nitrospira* MAGs. (A) Predicted urea metabolic pathways in acid-tolerant *Nitrospira* species based on MAGs retrieved in this study. (B) Phylogenetic tree of UT. Sequences from *Nitrospira* MAGs obtained in this study are shown in bold font with asterisks (^*^). Clusters composed of *Nitrospira* are indicated with shaded boxes. Bootstrap support from 500 iterations is shown by circles. The scale bar corresponds to 20% estimated sequence divergence. The outgroup, consisting of AOA, is indicated by an arrow. (C) Schematic illustration of the *Nitrospira* syntenic genomic regions containing UT genes. Inferred protein functions are indicated by color. The scale bar corresponds to 1 kb sequence length, and genes are drawn to scale. UrtABCDE, urea ABC-type transporter; UreABC, urease; UreEFGD, urease accessory proteins; UT, urea transporter; SMR, small multidrug-resistance protein; MFP, membrane fusion protein; RND, resistance-nodulation-division protein; OMF, outer membrane factor protein; OsmC, osmotically inducible protein C; MBL, metallo-β-lactamase.

AFB01 and NFB02 possessed urease, which catalyzes the hydrolysis of urea into ammonia and CO_2_ (see SI for detailed descriptions). Although the ammonia produced by urease can serve as a nitrogen source, it also functions as an acid neutralizer [75]. Since urease operates in the cytoplasm, urea is imported into cells by transporters. Two types of urea transporters were identified in AFB01 and NFB02 (Table S13): an adenosine triphosphate (ATP)-dependent ABC-type transporter (UrtABCDE) and ATP-independent passive transporters (UTs) (Fig. 5A).

The *urt* genes are present in all known urease-positive *Nitrospira* isolates, except *N. moscoviensis* [64]. Comammox *Nitrospira* enriched in a urine-fed bioreactor also retained these genes [76]. In contrast, ATP-independent UT-type transporters are rarely found in the genus *Nitrospira*, except in a few representatives (e.g., *N. japonica* [77], strain KM1 [78], CR1.3 [43], and CTRL-LIN-TMP-bin1 [67]). Accordingly, further investigation into *Nitrospira* UT was conducted.

UT forms homotrimers [79] and passively transports urea [80]. Urea imported into cells by UT is hydrolyzed by urease to produce free ammonia, which neutralizes acids (Fig. 5A). This UT-dependent acid-tolerance mechanism has been supported by studies on *Brucella melitensis* [81] and *Yersinia pseudotuberculosis* [82]. Although not conclusive, these studies suggest that UT may contribute to the acid tolerance of *Nitrospira*. It should be noted, however, that AFB01 and NFB02 may possess acid-tolerance mechanisms independent of urea, as these species were enriched in the absence of urea, similar to some acid-tolerant but nonureolytic ammonia oxidizers [83].

To investigate the phylogeny of *Nitrospira* UTs, a phylogenetic tree was constructed based on their amino acid sequences. As a result, *Nitrospira* UTs showed homology to those of acid-tolerant bacteria (e.g. *Yersinia*, *Brucella*, and *Klebsiella*) (Fig. 5B). Moreover, *Nitrospira* UTs branched into two clades. In this study, these clades are designated UT-1 and UT-2 for clarity. The single UT in AFB01 was classified into the UT-2 clade, whereas two copies in NFB02 belonged to separate clades (Fig. 5B).

Unlike UT, *Nitrospira* urease proteins (UreABC) form a monophyletic group [76]. To further investigate differences in their evolutionary pathways, the genomic contexts of the *ureABC* and UT genes were analyzed. As a result, the UT-1 gene was found in a cluster with genes for urease (*ureABCDEFG*) and ATP-dependent urea transporter (*urtABCDE*), whereas the UT-2 gene was located in a different context (Fig. 5C). Although further studies are required, this result suggests that UT-1 may have been inherited together with the *ure* and *urt* genes as a unit.

In contrast, UT-2 genes were clustered with genes involved in drug resistance and/or stress response (Fig. 5C), such as the small multidrug resistance (SMR) system [84], the resistance-nodulation-division (RND) efflux system consisting of the RND protein, the membrane fusion protein (MFP), and the outer membrane factor (OMF) protein [85], osmotically inducible protein C (OsmC) [86], metallo-β-lactamase (MBL) [87], and a sigma-54-dependent transcriptional regulator [88]. Given these genomic contexts, UT-2 may have been inherited not only for nitrogen metabolism but also for stress adaptation.

## Conclusion

This study characterized the physiological and genomic features of acid-tolerant comammox and NOB *Nitrospira*. Although it was difficult to determine which nitrifier contributed to nitrification under acidic conditions due to limitations of the experimental setup, physiological tests demonstrated that *Nitrospira* enrichment cultures retained nitrification activity after exposure to pH <5.5. The enriched comammox *Nitrospira* sp. AFB01 was phylogenetically close to clones derived from acidic soils, and its HAO active-site residues were identical to those of beta-AOB, suggesting that comammox *Nitrospira* contributes to nitrification in acidic soils and that HAO may be used as a common target for nitrification inhibitors in both comammox *Nitrospira* and beta-AOB. The key metabolic pathways of AFB01 and NFB02 were largely consistent with those of previously analyzed genomes. Notably, AFB01 showed high homology to a genome reconstructed from a neutral environment, suggesting that the enriched *Nitrospira* were acid-tolerant rather than acidophilic, and shared major metabolic features with species from nonacidic environments. Exceptionally, both MAGs possessed UTs homologous to those in acid-tolerant bacteria, which have been rarely found in the genus *Nitrospira*.

## Supplementary Material

Supplementary_information_ycaf167

Supplementary_datasets_ycaf167

## Data Availability

All data generated or analyzed during this study are included in this published article, its SI, and public databases. Raw metagenome sequencing data and MAGs of nitrifiers (AFB01, AFB05, AFB09, NFB01, and NFB02) were deposited in the DNA Data Bank of Japan, the European Nucleotide Archive, and the GenBank database under the accession number PRJDB12108.
