# Supplementary material for: Acid tolerance and metabolic potential of comammox and nitrite-oxidizing Nitrospira enriched from soil
Source: ISME Commun. 2025 Sep 23;5(1):ycaf167. doi: 10.1093/ismeco/ycaf167 (PMC12516955; doi:10.1093/ismeco/ycaf167)
Supplement: Supplementary_information_ycaf167 [file supplementary_information_ycaf167.pdf]

Supplementary Information for:

Acid tolerance and metabolic potential of comammox and nitrite-oxidizing *Nitrospira* enriched from soil

Yu Takahashi[1], Hirotsugu Fujitani[2,3], Itsuki Taniguchi[4], Yasuhiro Gotoh[5], Yuta Shimada[1], Shuto Ikeda[1], Tetsuya Hayashi[4], Kanako Tago[6,7], Masahito Hayatsu[7], Satoshi Tsuneda[1]

1) Department of Life Science and Medical Bioscience, School of Advanced Science and Engineering, Waseda University, Shinjuku-ku, Tokyo, Japan

2) Department of Biological Sciences, Faculty of Science and Engineering, Chuo University, Bunkyo-ku, Tokyo, Japan

3) Department of Applied Physics and Chemical Engineering, Faculty of Engineering, Tokyo University of Agriculture and Technology, Koganei-shi, Tokyo, Japan

4) Department of Bacteriology, Graduate School of Medical Sciences, Kyushu University, Fukuoka-shi, Fukuoka, Japan

5) Advanced Genomics Center, National Institute of Genetics, Mishima-shi, Shizuoka, Japan

6) Department of Green Innovation and Environmental Bioscience, School of Veterinary Medicine, Kitasato University, Sagamihara-shi, Kanagawa, Japan

7) Institute for Agro-Environmental Sciences, National Agriculture and Food Research Organization (NARO), Tsukuba-shi, Ibaraki, Japan

Corresponding author: Satoshi Tsuneda, Department of Life Science and Medical Bioscience, School of Advanced Science and Engineering, Waseda University, 2-2 Wakamatsu-cho, Shinjuku-ku, Tokyo, 162-8480 Japan; E-mail address: stsuneda@waseda.jp

Supplementary Materials and Methods

Acid tolerance evaluation of AFB *Nitrospira* enrichment

Inorganic medium (**Table S1**) and 1.70 g/L KH_2PO_4 solution were prepared with Milli-Q water, sterilized by autoclaving, and mixed at 50:1 (v/v). The mixture was filtered through a polyethersulfone filter unit VFTB-500 with a pore size of 0.22 µm (AS ONE, Osaka, Japan) and its pH was adjusted to approximately 6 using HCl and NaOH solutions.

From the ammonium-fed bioreactor (AFB) operated in our previous study [1], 20 mL of *Nitrospira* enrichment culture was collected. The enrichment was divided into two tubes and centrifuged (2,900 xg, 5 min). The supernatants were removed from the tubes. The tubes were added with 5 mL/tube of fresh medium and placed on ice. The pellets in tubes were dispersed using a Q55 sonicator (Qsonica LLC, Newtown, PA, USA) for 20 s intermittently at an amplitude of 20%. Centrifugation, supernatant removal, medium addition, and dispersion were repeated to wash the supernatant from AFB. The washed bacterial cells were suspended in 10 mL of a fresh medium. A 300 mL of fresh medium and 10 mL cell suspension were mixed in a 500 mL glass bottle with screw cap. An autoclaved NH_4Cl solution was added to this bottle at a final concentration of 5 mM. The above procedures were conducted in biological duplicate, and the two bottles were designated bottle 1 and bottle 2.

The bottles with AFB enrichment were incubated statically in the dark at room temperature (23°C). The pH of the medium (pH_{medium}) was measured every few days using a pH meter LAQUAtwin (Horiba, Kyoto, Japan). Because the pH_{medium} decreases due to nitrification, 500 µL of an autoclaved 50 g/L $NaHCO_3$ solution was added to the bottles on days 7, 13, and 25 to increase the pH_{medium} to 4.28–5.92. The culture was sampled on days 0, 7, 10, 13, 20, 25, 26, and 28. The samples were stored at −20°C until the measurement of total ammonia, nitrite, and nitrate concentrations. The bacterial cells in samples were precipitated by centrifugation (13,000 xg, 5 min), and the supernatant was used for the measurement.

After 28 days of incubation, 160 mL of culture from bottle 2 was collected. The enrichment was divided into four tubes and centrifuged (2,900 xg, 5 min). The supernatants were removed, and 5

mL of fresh medium was added to each tube. The pellets were resuspended by vortexing. A 300 mL of fresh medium and 20 mL of cell suspension were combined in a 500 mL screw-cap glass bottle. An autoclaved NH_4Cl solution was then added at a final concentration of 5 mM. This bottle was designated bottle 3. Static incubation, pH_{medium} measurement, and sampling were performed using the same method for bottle 2. On day 12 of incubation, 500 µL of an autoclaved 50 g/L $NaHCO_3$ solution was added to increase the pH_{medium} to 6.77. The culture was sampled on days 0, 4, 6, 12, 18, and 24.

Test for recovery from acidic stress of NFB *Nitrospira* enrichment

Inorganic medium (**Table S2**) and 3.82 g/L K_2HPO_4 solution were prepared with Milli-Q water, sterilized by autoclaving, and mixed at 100:1 (v/v). The mixture was filtered through a polyethersulfone filter in the same method as medium for AFB. The pH_{medium} was adjusted to approximately 8 using HCl and NaOH solutions.

From the nitrite-fed bioreactor (NFB) operated in our previous study [1], 70 mL of *Nitrospira* enrichment culture was collected. The enrichment was divided into two tubes. Centrifugation, supernatant removal, medium addition, and dispersion were conducted two times with the same method for AFB. The washed bacterial cells were suspended in 10 mL/tube of a fresh medium at pH 8.

Into the fresh medium described above, 2-morpholinoethanesulfonic acid (MES) was added at a final concentration of 80 mM as a pH buffer. The buffered pH_{medium} was adjusted to pH 3.3 and pH 2.4 using HCl and NaOH solutions. The medium at pH 3.3 and 2.4 was transferred to a 500 mL glass bottle with screw cap at 150 mL/bottle. Into the bottles, the washed NFB cell suspension was added at 10 mL/bottle. NFB enrichment exposed to acidity at pH 3.3 or 2.4 was incubated statically in the dark at room temperature (23°C) for seven days. During the acid exposure, the pH_{medium} was measured daily using a pH meter LAQUAtwin (Horiba).

After seven days of acid exposure, 40 mL/bottle of *Nitrospira* enrichment cultures were collected and centrifuged (2,900 xg, 5 min). The supernatants were removed from the tubes. The tubes were added with 10 mL/tube of fresh medium without MES and placed on ice. The pellets in tubes

were dispersed as described above. Centrifugation and supernatant removal, medium addition, and dispersion were repeated to wash the supernatant from acidic medium with MES. The washed bacterial cells were suspended in 5 mL/tube of a fresh medium at pH 8. The suspension was transferred into three glass test tubes at 1 mL/tube (in biological triplicate). A fresh medium was added to adjust the volume to 10 mL. An autoclaved $NaNO_2$ solution was added at a final concentration of 720 μM.

The NFB enrichment cultures were incubated statically in the dark at room temperature (23°C). On days 0, 2, 3, 6, 17, 28, and 35, 200 μL of the medium was sampled and stored at −20°C until the measurement of nitrite concentrations. The samples were thawed and filtered through polytetrafluoroethylene (PTFE) syringe filters with a pore size of 0.22 μm (AS ONE), and the supernatant was used for the measurement.

Mean nitrite consumption rates were calculated based on the initial nitrite concentrations and the number of days required for complete consumption (28 or 35 days at pH 2.4; 6 days at pH 3.3). The normality of the data was assessed using the Kolmogorov–Smirnov test, which indicated that the data were normally distributed (data not shown). Consequently, an *F*-test was performed to assess the equality of variances between the two groups, "from pH 2.4 to pH 8" and "from pH 3.3 to pH 8", and the variances were found to be unequal (data not shown). Therefore, Welch's two-sided *t*-test with an alpha level of 0.05 was used to compare the mean nitrite consumption rates between the two groups.

Test for nitrification rates of AFB enrichment at different ammonia concentrations

From AFB, 40 mL of enrichment culture was collected. Preparation of the inorganic medium, centrifugation, supernatant removal, medium addition, and dispersion were performed in the same manner as described in "Acid tolerance evaluation of AFB *Nitrospira* enrichment."

The washed bacterial cells were resuspended in 10 mL of fresh medium containing 0, 0.5, 1.0, 2.5, 5.0, or 10 mM NH_4Cl in glass tubes. This procedure was conducted in biological triplicate. The tubes were incubated statically in the dark at 28 °C for five days. One milliliter of culture was sampled from each tube on days 0, 1, 2, and 5. Of this, 500 μL was used to measure the pH_{medium} with a pH meter LAQUAtwin (Horiba), and the remaining samples were stored at −20°C until nitrate

concentrations were measured.

Test for nitrification rates of NFB enrichment at different nitrite concentrations

Preparation of the inorganic medium, collection of enrichment culture from NFB, centrifugation, removal of supernatant, medium addition, and dispersion were performed as described in "Test for recovery from acidic stress of NFB *Nitrospira* enrichment." Exceptionally, the medium was unbuffered, and its pH_{medium} was adjusted to 7.2.

The cell suspension and fresh medium were mixed in glass tubes at a final volume of 30 mL per tube, and the final nitrite concentrations in the medium were adjusted to 0, 0.71, 3.6, 7.1, 21, 36, or 71 mM. This procedure was conducted in biological triplicate. The tubes were incubated statically in the dark at room temperature (23°C) for four days. One milliliter of culture was sampled from each tube daily and stored at −20°C. The samples were thawed and filtered through syringe filters as described above, and the supernatant was used for measuring nitrate concentrations.

Genomic DNA extraction

From AFB and NFB, enrichment cultures of 10 mL each were collected. The samples were centrifuged (2,900 xg, 5 minutes) and supernatants were removed. The pellets were resuspended in 100 µL of TE buffer and dispersed using a Q55 sonicator (Qsonica LLC) for 10 s intermittently at an amplitude of 10%. The suspensions of 100 µL, 0.3 g of zirconia balls YTZ-0.1 (Nikkato, Osaka, Japan), and 150 µL of MagNA Pure Bacteria Lysis Buffer (Roche Diagnostics, Indianapolis, IN, USA) were added into tubes TM-625S (TOMY, Tokyo, Japan). The tubes were vortexed and incubated at 37°C for 15 minutes. During the incubation, tubes were vortexed every 5 minutes. Liquid Proteinase K of 200 µL and Lysis Buffer H1 of 150 mL included in the NucleoBond HMW DNA kit (Macherey-Nagel, Düren, Germany) were added to the tubes. The mixture was vortexed and incubated at 37°C for 15 minutes. During the incubation, the tubes were vortexed every 5 minutes. The cells were disrupted using a Bead Smash 12 (Wakenyaku, Kyoto, Japan) at 5,500 rpm for 5 minutes. The samples were centrifuged (11,000 x g, 5 minutes, room temperature) and supernatants were transferred to new 50 mL centrifuge tubes. The

tubes were added with 4 mL/tube Lysis Buffer H1, vortexed for 5 s, and incubated at 50°C for 30 minutes. During the incubation, the tubes were inverted every 5 minutes to mix. Genomic DNA was extracted from the lysates according to the standard protocol 'Bead beating based cell lysis' of the NucleoBond HMW DNA kit (Macherey-Nagel). After ethanol precipitation and washing, DNA was eluted into elution buffer overnight.

Metagenomic sequencing and hybrid assembly

DNA libraries were prepared using the NEBNext Ultra II FS DNA Library Prep Kit for Illumina (New England Biolabs, Ipswich, MA, USA) and the Rapid Barcoding Sequencing kit (SQK-RBK004; Oxford Nanopore Technologies, Oxford, UK) and sequenced using HiSeq (Illumina, San Diego, CA, USA) and MinION (Oxford Nanopore Technologies), respectively. HiSeq pair-end short reads (150 bp × 2) were trimmed using platanus_trim version 1.0.7 [2] (quality 15, length 25). Random sampling was performed by SeqKit version 0.15.0 [3] (-p 0.66, -s 11) for short reads obtained from AFB. MinION long reads were trimmed using NanoFilt version 2.8.0 [4] (-l 2000, -q 10, --headcrop 100). Hybrid assembly was performed with OPERA-MS version 0.9.0 [5] and binned by MetaBAT2 version 2.15 [6]. Completeness, contamination, and strain heterogeneity of reconstructed metagenome-assembled genomes (MAGs) were calculated with CheckM version 1.1.2 [7].

To minimize undesirable noises caused in assembly using excessive numbers of reads, various numbers of HiSeq and MinION reads were used with different combinations in assembly. For HiSeq reads, 100% of the filtered reads and 5%, 10%, and 20% of the filtered reads, which were randomly extracted, were used. For MinION reads, read sets extracted with the 2, 7, 10, or 15 kbp read length threshold were used. All combinations were tested, and the combinations that generated the highest quality of *Nitrospira* MAGs were selected in terms of the number of sequences (contigs), contamination, and completeness. Consequently, 100% HiSeq reads and >7 kbp MinION reads were used for the AFB assembly and 20% HiSeq reads and >7 kbp MinION reads were used for the NFB assembly (**Table S3**).

According to a previous report [8], MAGs with high completeness (≥90%) and low

contamination (≤10%) are high-quality MAGs and were used for subsequent analyses. The relative abundance of MAGs was calculated by mapping HiSeq reads (**Table S3**) via CoverM version 0.7.0 [9] (--min-read-aligned-percent 0.75, --min-read-percent-identity 0.95, and --min-covered-fraction 0).

Quantification of AFB01-related sequences in soils

Raw comammox *amoA* amplicon sequences obtained from agricultural soils in a previous study [10] (accession numbers: DRR400646–DRR400663) were downloaded from the NCBI Sequence Read Archive (SRA). FASTQ-format files were extracted from SRA files using the fasterq-dump tool in SRA-toolkit version 3.2.1 (SRA Toolkit Development Team, https://trace.ncbi.nlm.nih.gov/Traces/sra/sra.cgi?view=software).

Following the DADA2 ITS Pipeline Workflow (1.8) (https://benjjneb.github.io/dada2/ITS_workflow.html), primer sequences in the raw amplicon sequences were identified using DADA2 version 1.37.0 [11], ShortRead version 1.67.0 [12], and Biostrings version 2.77.1 [13] in R version 4.5.0 [14], and were subsequently removed using Cutadapt version 4.0 [15]. Primer-trimmed reads were filtered and trimmed using DADA2 with standard parameters and minimum length threshold of 150 bps. Paired reads were merged, a sequence table was constructed, and chimeras were removed using DADA2 with default settings to generate comammox *amoA* amplicon sequence variants (ASVs).

The resulting ASVs were compared to the *amoA* gene sequence of AFB01 (locus tag: NTAFB01_16080) using the BLASTn tool from BLAST+ version 2.16.0 [16]. ASVs producing significant alignments to AFB01 *amoA* were identified, and the aligned ASVs sharing >98% and >90% nucleotide identity with AFB01 *amoA* were quantified. The means and standard deviations of relative abundances were calculated from technical triplicates, as soil sampling and amplicon sequencing were performed in technical triplicates in the original study [10].

Comparative genomics

The amino acid sequences from *Candidatus* Nitrospira kreftii (ASM1405840v1), *Candidatus*

Nitrospira nitrificans (GCA_001458775.1), *Candidatus* Nitrospira nitrosa (GCA_001458735.1), *Nitrospira inopinata* (GCA_001458695.1), *Nitrospira japonica* NJ1 (GCA_900169565.1), *Nitrospira* sp. KM1 (ASM1140551v1), and *Nitrospira moscoviensis* NSP M-1 (ASM127377v1) were retrieved from the NCBI Assembly database [17] as faa files, with the accession numbers shown in parentheses.

The amino acid sequences of all proteins from AFB01, *Ca.* N. kreftii, *Ca.* N. nitrificans, *Ca.* N. nitrosa, and *N. inopinata* were analyzed using OrthoVenn2 [18] with the default parameters to identify orthologous proteins. Proteins without orthologs in the other species were classified as singletons, and the singletons of AFB01 were regarded as its unique proteins.

Similarly, the same analysis was performed for NFB02 together with *N. japonica* NJ1, *N.* sp. KM1, and *N. moscoviensis* NSP M-1, and the singletons of NFB02 were regarded as its unique proteins.

Supplementary Results and Discussion for Physiological Tests

Test for nitrification rates of AFB enrichment at different ammonia concentrations

To assess nitrification rates under varying initial total ammonia concentrations, washed bacterial cells from AFB enrichment were incubated. During the first two days, nitrate concentrations showed almost no differences across ammonia concentrations (Fig. S8A). However, a clear difference in nitrate concentrations became apparent on day 5, depending on the initial ammonia concentration (Fig. S8A). Over the five-day period, the pH_{medium} consistently decreased (Fig. S8B). As a result, the mean nitrate production rate during these five days peaked at 108 ± 10.1 μM/day under the 5 mM ammonia condition (Fig. S8C). It should be noted that nitrate production was also observed at 0 mM initial ammonia (Fig. S8A and S8C).

The slow nitrate production rates, especially during the first two days, may have been due to the small amount of biomass used in the physiological experiments. Biomass in AFB was limited because of the low growth rate of comammox *Nitrospira* under the harsh condition of pH 5.5, which made it difficult to use sufficient biomass in the physiological tests. However, collecting larger amounts of biomass may have been necessary to obtain clearer results in short-term incubations and to minimize the influence of cell growth as well as changes in bacterial community structure during the experiments. Furthermore, the nitrate production at 0 mM ammonia was likely due to the mineralization of organic nitrogen compounds released from the cells, resulting in ammonia generation in the medium.

Test for nitrification rates of NFB enrichment at different nitrite concentrations

To assess nitrite oxidation rates under varying initial nitrite concentrations, washed bacterial cells from NFB enrichment were incubated. During the first three days of incubation, nitrate concentrations consistently increased at 0.71 and 3.6 mM nitrite (Fig. S9A). In contrast, at 7.1 mM nitrite, nitrate production was not apparent during this period, but the nitrate concentration markedly increased on day 4 (Fig. S9B). Nitrate concentrations remained low on day 4 at 21–71 mM of nitrite (Fig. S9B). As a result, mean nitrate production rates over the four days peaked at 166 ± 4.57 μM/day at 0.71 mM

nitrite and decreased at ≥21 mM nitrite (Fig. S9C).

As nitrate production at 7.1 mM nitrite was not apparent during the first three days (Fig. S9A), the mean nitrate production rates were calculated over the entire four-day incubation period. However, nitrate concentrations did not increase during days 3–4 of incubation at 3.6 mM nitrite (Fig. S9A). Moreover, the nitrate production rates of biological triplicates showed considerable variation at 3.6 and 7.1 mM nitrite, resulting in large standard deviations (Fig. S9C). For these reasons, this experiment could not clearly demonstrate the relationship between initial nitrite concentrations and nitrate production rates of NFB enrichment. Similar to the case of AFB, collecting larger amounts of biomass and appropriately adjusting the incubation time were required to resolve this issue.

Supplementary Results and Discussion for Metabolic Pathway Annotation

Nitrification

AFB01 contained genes for ammonia monooxygenase (AMO) subunits (*amoCAB*) and the hydroylamine:ubiquinone reduction module (HURM) consisting of *haoAB* and *cycAB* (**Table S13**; **Dataset S1**). AMO and HURM are included in the key enzymes of bacterial ammonia oxidation [19]. Furthermore, a copper-containing nitrite reductase (NirK) was commonly preserved in AFB01 and NFB02 (**Table S13**). It is hypothesized that NirK catalyzes the final step of ammonia oxidation of AOB, conversion of nitric oxide (NO) into nitrite [20]. Although not validated, it has been speculated that a common ammonia oxidation pathway is conserved between AOB and comammox *Nitrospira*, because they share homologous AMO, HAO, and NirK [21].

Both AFB01 and NFB02 had all the genes for nitrite oxidoreductase (NXR) subunits (*nxrABC*) (**Table S13**; **Dataset S1**). NXR is the key enzyme for nitrite oxidation of NOB and comammox bacteria [22]. In conclusion, AFB01 and NFB02 possessed genes required for complete ammonia oxidation and nitrite oxidation, respectively.

Nitrogen assimilation

Both AFB01 and NFB02 lacked the assimilatory ferredoxin-dependent nitrite reductase (NirA), an assimilatory nitrite-reducing enzyme (**Table S13**). However, NFB02 possessed an octaheme cytochrome *c* (OCC) and a transmembrane Rieske/cytochrome *b* complex (**Table S13**; **Dataset S1**). As predicted in *Nitrospira moscoviensis* [23] and *Nitrospira japonica* [24], NFB02 may reduce nitrite to ammonia via OCC for nitrogen assimilation. In contrast, AFB01 lacked both nitrite reductases. Thus, AFB01 may be unable to grow on nitrite as a sole nitrogen source as well as *N. inopinata* [25].

To transport ammonia for nitrogen assimilation, AFB01 had a Rh-type transporter (**Table S13**), which was consistent with previously reported comammox clade A members [19]. NFB02 had two copies of Amt-type transporter (**Table S13**; **Dataset S1**) and multiple Amt homologs, which was similar to *N. moscoviensis* [23].

In addition to external ammonium, some *Nitrospira* utilize cyanate and/or urea as nitrogen

sources [19]. Both AFB01 and NFB02 preserved the cyanate hydratase gene *cynS* (**Table S13**; **Dataset S1**), suggesting their potential to generate ammonia and CO_2 from cyanate intracellularly. The cyanate degradation of *N. moscoviensis* has been experimentally demonstrated [26]. Furthermore, *cynS*-encoding comammox *Nitrospira* metagenomes have been reconstructed from wastewater treatment plants (WWTPs) [27] and rotating biological contactors [28].

Urea degradation

NFB02 possessed all the genes for nickel (Ni)-dependent urease (UreABC) and urease accessory proteins (UreDEFG) (**Table S13**; **Dataset S1**). AFB01 also had all the genes for urease enzymes and accessary proteins, except for *ureE* gene (**Table S13**; **Dataset S1**). Likewise, *N. moscoviensis* had no functional UreE protein, but it was able to degrade urea [23]. In this organism, the function of UreE was speculated to be substituted by the (NiFe)-hydrogenase maturation enzymes HypA and HypB [23]. Although AFB01 lacked *hypB* gene, it had *hypA* gene (**Dataset S1**) which may help ureolysis as proposed in *N. moscoviensis*.

Energy and carbon metabolisms

The previously analyzed *Nitrospira* genomes commonly included the genes for five complexes of the respiratory chain, the reductive and oxidative TCA (tricarboxylic acid) cycle, gluconeogenesis, and the pentose phosphate cycle [19]. AFB01 and NFB02 preserved almost all the genes for these pathways (**Table S13**). However, some pathways were incomplete; AFB01 lacked the genes encoding phosphoenolpyruvate carboxylase of the reductive TCA cycle; NFB02 lacked the genes encoding QcrC constituting complex III of the respiratory chain, pyruvate carboxylase subunit A of the reductive TCA cycle, and phosphoenolpyruvate carboxykinase of the gluconeogenesis pathway (**Table S13**; **Dataset S1**). The absence of these key genes in AFB01 and NFB02 might be because of incompleteness of the assemblies (**Table S7**).

Formate metabolism

NFB02 possessed genes for a formate transporter and the three subunits of formate dehydrogenase FdsABG, required for formate-dependent growth, while AFB01 lacked these genes (**Table S13, Dataset S1**). This was consistent with previous reports because this gene cluster was identified in NOB and clade B comammox *Nitrospira* but was not in clade A [29].

However, AFB01 had genes of a molybdenum-dependent formate dehydrogenase (FdhF) and an accessory sulfurtransferase (FdhD) (**Table S13**; **Dataset S1**). This preservation was consistent with clade A comammox *Nitrospira* sp. LK70, a metagenome reconstructed from a WWTP [27]. These results suggested that both AFB01and NFB02 may utilize formate as a source for carbon and energy.

Hydrogen metabolism

AFB01 had a [NiFe]-hydrogenase group 3b (**Table S13**; **Dataset S1**). The function of this protein in comammox *Nitrospira* is still in discussion [19]. Although not an identical protein, a group 2a [NiFe]-hydrogenase was possessed in *N. moscoviensis*, and the strain grew aerobically with hydrogen as a sole energy source [30]. Furthermore, AFB01 possessed some genes for hydrogenase maturation (**Table S13, Dataset S1**). The genes for group 3b hydrogenase and maturation proteins were also found in clade A comammox *Nitrospira* sp. WS110, reconstructed from a WWTP [27].

Superoxide detoxification

AFB01 had superoxide dismutase (SOD) and catalase (KatA) for defense against reactive oxygen species (**Table S13**; **Dataset S1**). SOD catalyzes the dismutation of superoxide into hydrogen peroxide and oxygen, and catalase converts hydrogen peroxide to oxygen and water [31].

Contrary, NFB02 had two types of SODs but no catalase (**Table S13**; **Dataset S1**). *Nitrospira defluvii* also lacked catalase, but its cytochrome *c* peroxidases and thioredoxin-dependent peroxiredoxins were speculated to function as H_2O_2 scavengers [32]. Likely, NFB02 possessed cytochrome c_{551} peroxidase (locus tag: NTNFB02_15660), thioredoxins (locus tag: NTNFB02_01350, NTNFB02_10630, NTNFB02_30610, and NTNFB02_36910), and their reductases (locus tag:

NTNFB02_08770 and NTNFB02_25500).

Multiple resistance and pH adaptation (Mrp) cation/proton antiporter

AFB01 and NFB02 possessed genes for Mrp antiporter (**Table S13**). Mrp (alias Mnh and Pha) functions as a cation: proton antiporter. Mrp of *Sinorhizobium* (formerly *Rhizobium*) *meliloti* functions as a K^+ (Na^+)/H^+ antiporter and contributed to potassium-dependent alkaline pH homeostasis ability [33]. Mrp of *Bacillus subtilis*, contributed to Na^+ homeostasis/tolerance [34].

The subunits of Mrp, MrpABCDEFG, in *Bacillus subtilis* were suggested to form a complex to function as Na^+/H^+ antiporter [35]. AFB01 and NFB02 commonly had *mrpBCDEFG*, and their *mrpB* genes contained fused sequences of *mrpA* and *mrpB* (**Dataset S1**). Such contexts of *mrp* genes are classified into group 2 *mrp* operon consisting of six genes [36].

Acid tolerance

The repertoires of homologs of acid tolerance-related proteins in AFB01 and NFB02 were largely consistent with those found in *Ca.* Nitrospira kreftii and *N. japonica* (**Table S14**). This result refutes the idea that well-known acid tolerance mechanisms are uniquely preserved in AFB01 and NFB02.

The presence of similar acid tolerance-related proteins in both acid-tolerant and non-acid-tolerant *Nitrospira* spp. may reflect functions of the query proteins unrelated to acid tolerance. Overall, these proteins are involved in major cellular processes such as central carbon metabolism, amino acid metabolism, energy production, cell membrane construction, and cellular homeostasis. For example, cyclopropane fatty acyl phospholipid synthase (Cfa) contributes to the synthesis of cyclopropane fatty acids in cell membrane phospholipids [37]. Glycine betaine transporter (BetL) facilitates the accumulation of betaine [38]. Glutamyl cysteine synthetase (GshA) and glutathione synthetase (GshB) are involved in glutathione biosynthesis [39]. Trehalose 6-phosphate phosphorylase (TrePP), beta-phosphoglucomutase (PgmB), and trehalose 6-phosphate phosphatase (OtsB) are associated with trehalose synthesis [40]. Histidine decarboxylase (HdcA), its maturation protein (HdcB), and the histidine/histamine antiporter (HdcP) contribute to proton consumption via amino acid

decarboxylation [41]. Glutaminase (YbaS) converts L-glutamine into L-glutamate and free ammonia, while the glutamate:gamma-aminobutyrate antiporter (GadC) exchanges extracellular L-glutamine with intracellular L-glutamate [42]. Undecaprenyldiphospho-muramoylpentapeptide beta-N-acetylglucosaminyltransferase (MurG) participates in peptidoglycan biosynthesis [43]. The acid-activated periplasmic chaperones HdeAB and their cytoplasmic counterpart Hsp31 refold proteins denatured by acid stress [44]. ATP synthase subunit alpha (AtpA) supports acid tolerance through energy generation [45]. Carbonic anhydrase (CA) catalyzes the reversible hydration of CO_2 [46].

AFB01 possessed homologs of TrePP, PgmB, and OtsB (**Table S14**). This finding supports the possibility that AFB01 can convert glucose-6-phosphate into trehalose, which is known to protect cells from a variety of environmental stresses [40]. However, this mechanism may not be essential for acid tolerance in *Nitrospira*, as NFB02 lacks TrePP.

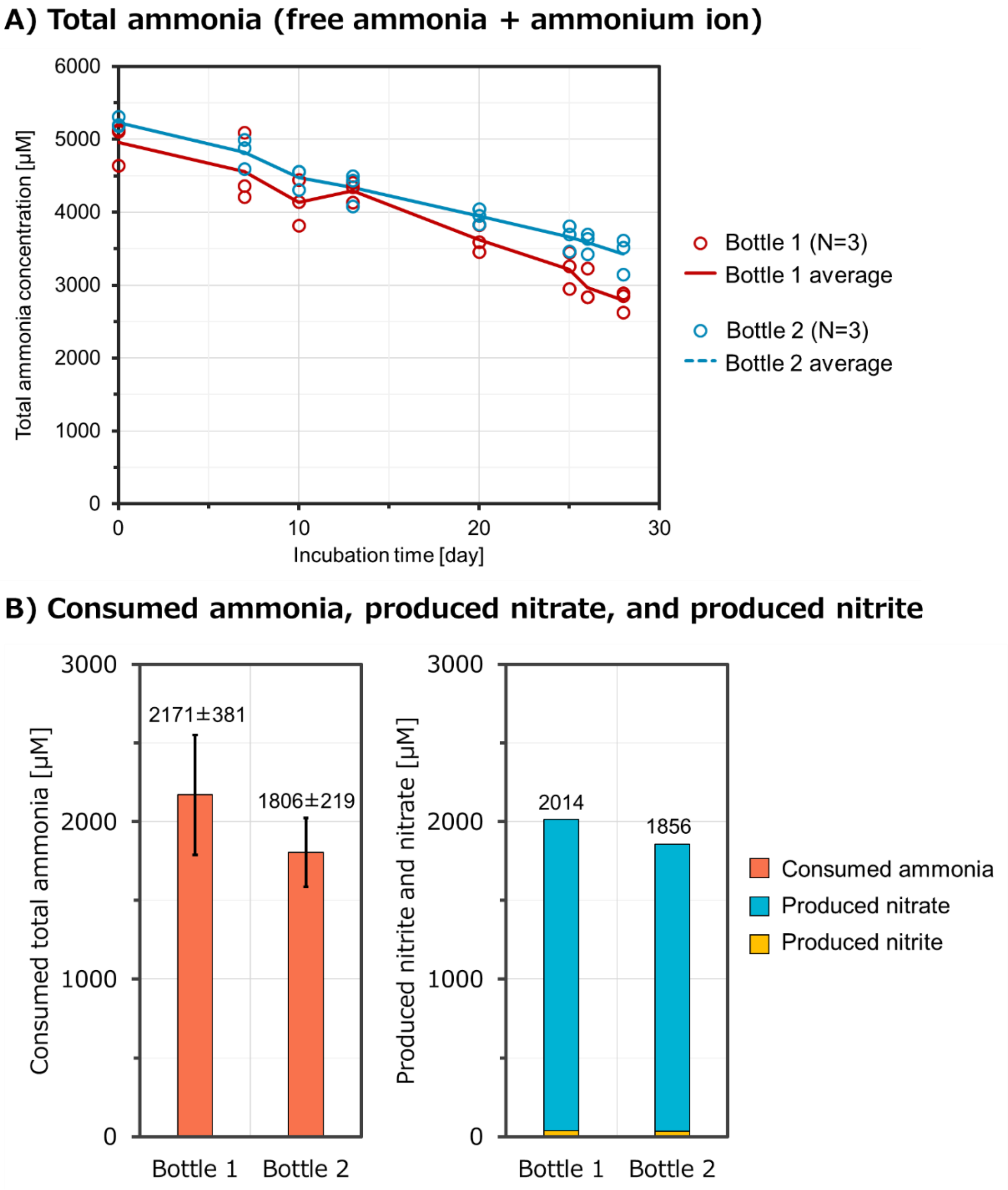

Fig. S1. (A) Total ammonia concentrations in the medium during the cultivation of AFB enrichment, conducted in biological duplicates **(**red, bottle 1; cyan, bottle 2). Circles represent values measured in technical triplicate, and solid lines indicate the averages of these triplicates. **(B)** Concentrations of consumed total ammonia (orange), produced nitrate (cyan), and produced nitrite (yellow) during the cultivation of AFB enrichment. Error bars for consumed total ammonia represent the standard deviations of technical triplicates.

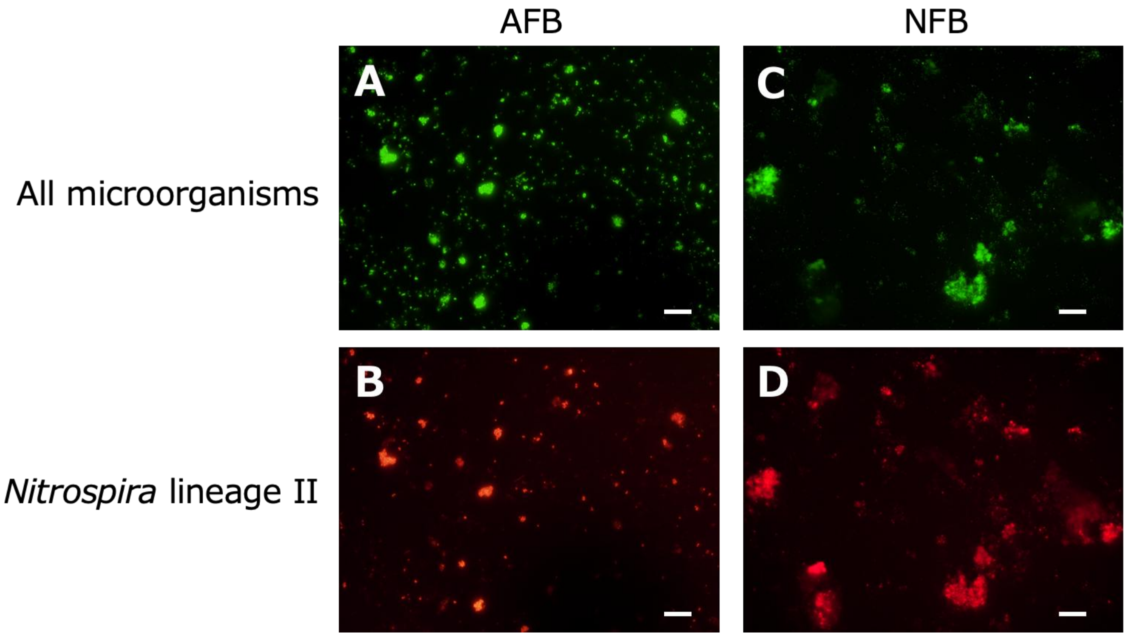

Fig. S2. Micrographs of **(A, B)** half-batch culture of AFB incubated for 26 days, and **(C, D)** culture of NFB exposed to acidic condition at pH 2.4 for a week. **(A, C)** All microorganisms were labeled with SYTOX Green. **(B, D)** Only *Nitrospira* lineage II cells were labeled with fluorescence *in situ* hybridization (FISH) probes. The scale bars represent 10 µm.

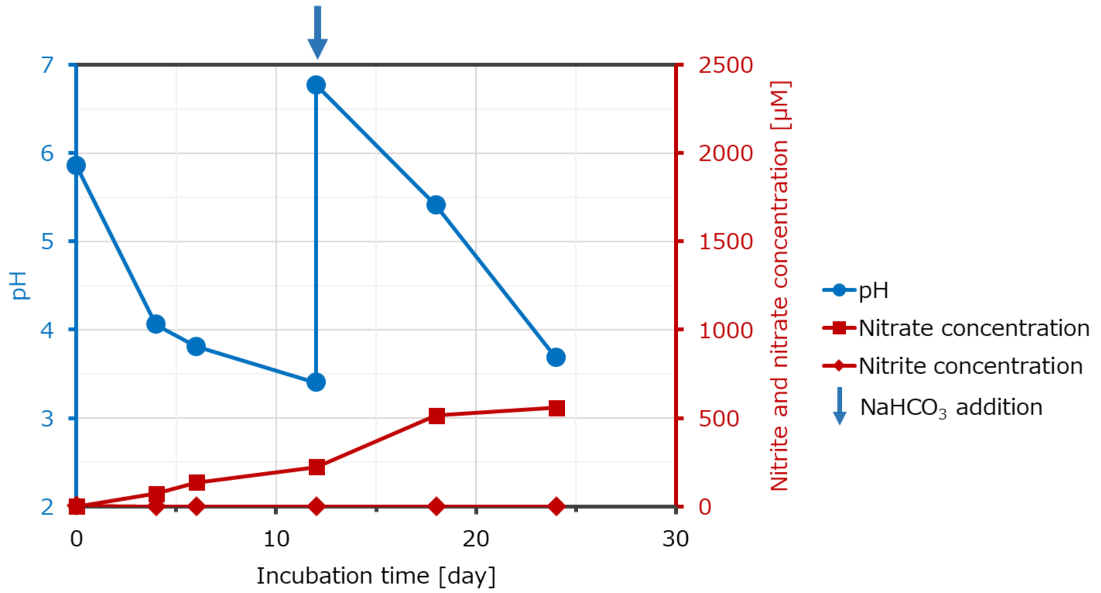

Fig. S3. Ammonia oxidation by AFB enrichment in bottle 3. The plots show pH (blue circles), nitrate concentrations (red squares), and nitrite concentrations (red diamonds) in the medium. The vertical blue arrow indicates the timing of $NaHCO_3$ supplementation on day 12 of incubation.

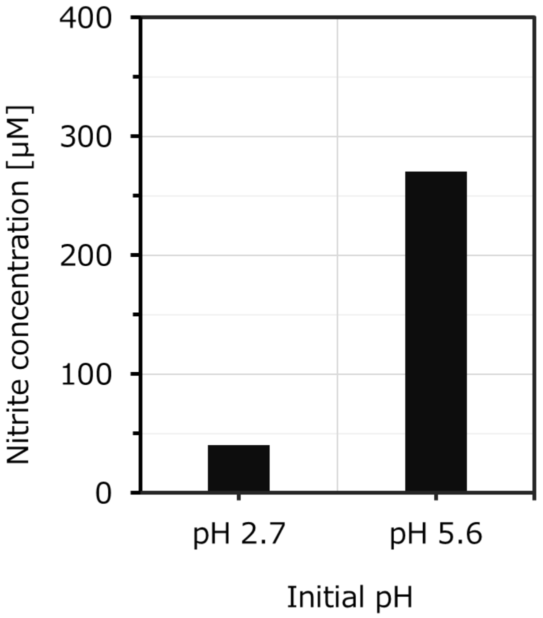

Fig. S4. Nitrite concentrations after two days of spontaneous degradation.

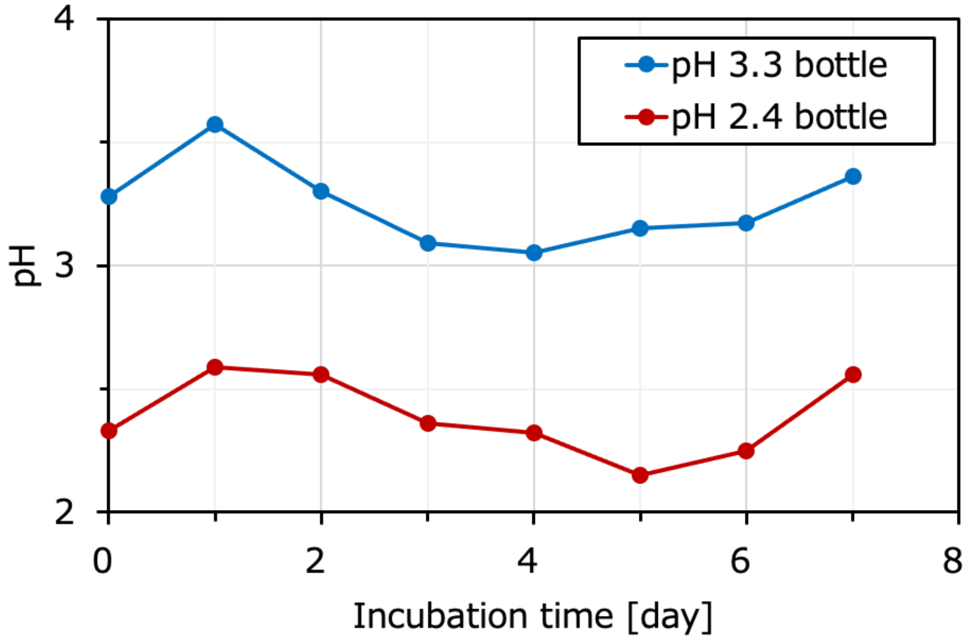

Fig. S5. The pH_{medium} values during acid exposure (red, pH 2.4 bottle; blue, pH 3.3 bottle) in the physiological test of NFB *Nitrospira* enrichment.

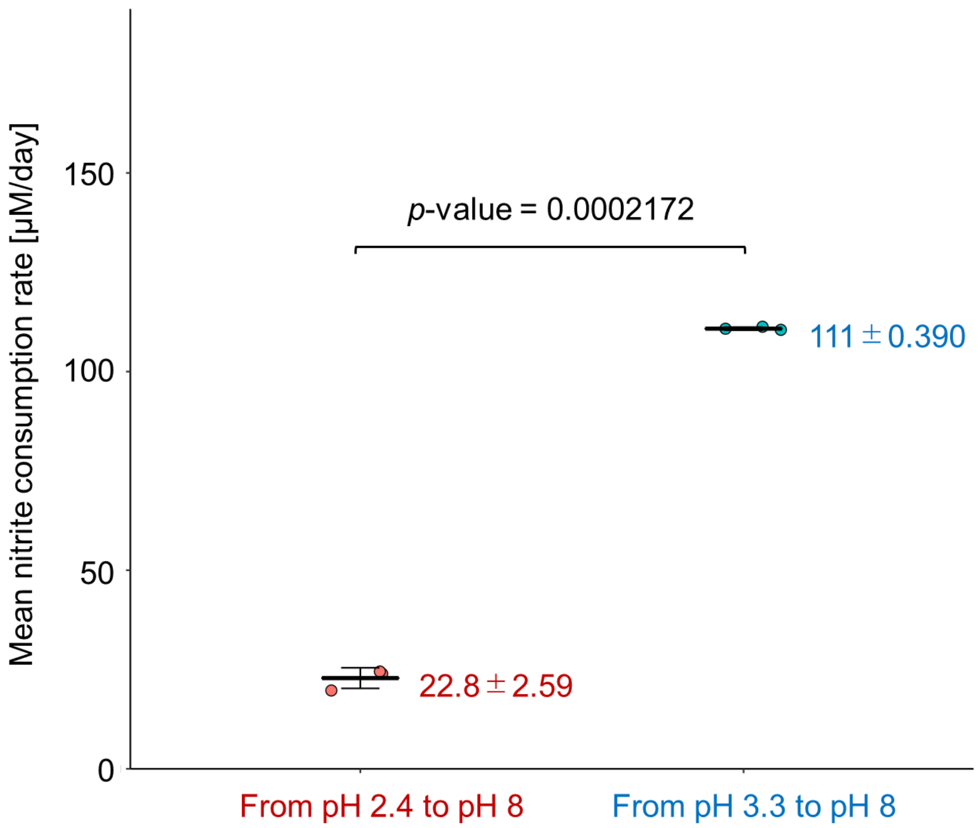

Fig. S6. Mean nitrite consumption rates during cultivation of NFB enrichment after exposure to acidity at pH 2.4 (red) and 3.3 (blue). Rates were calculated based on the initial nitrite concentrations and the number of days required for complete consumption (28 or 35 days at pH 2.4; 6 days at pH 3.3). Values represent the means ± standard deviations of biological triplicates. Horizontal bold lines indicate means, and thin lines indicate standard deviations. The *p*-value was calculated using Welch's *t*-test (two-sided) with an alpha level of 0.05.

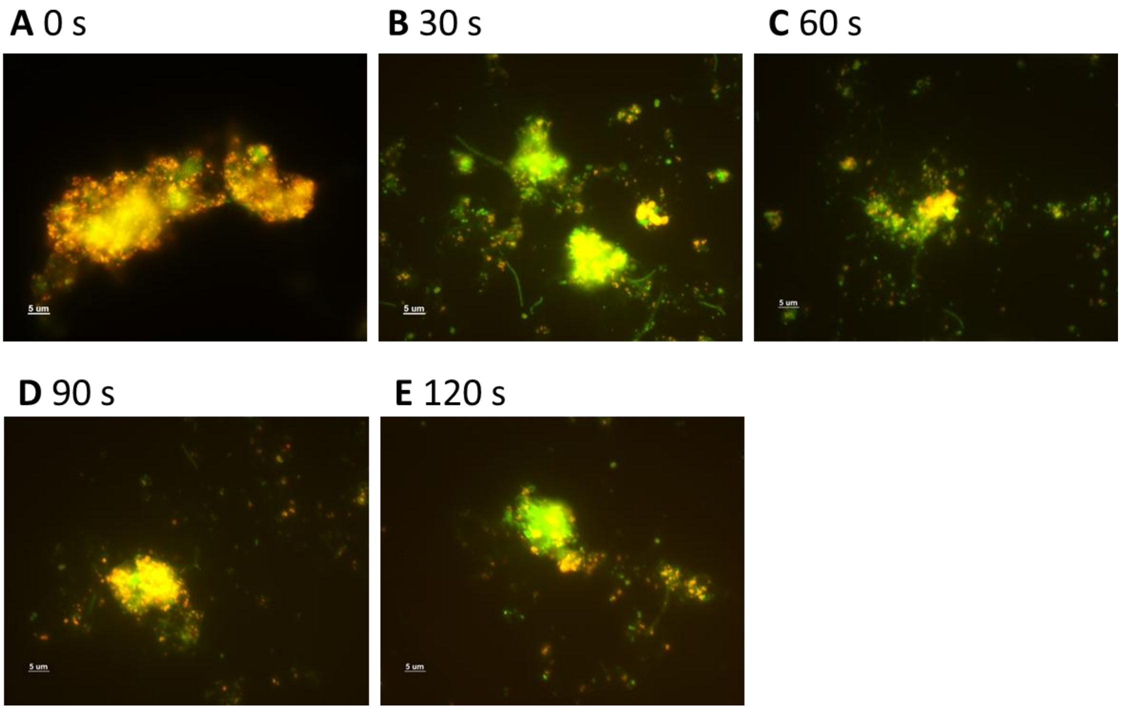

Fig. S7. Micrographs of NFB enrichment dispersed by sonication for **(A)** 0s, **(B)** 30s, **(C)** 60 s, **(D)** 90 s, and **(E)** 120 s. Scale bars represent 5 μm. All microorganisms were stained with SYTOX Green (green), while *Nitrospira* lineage II cells were labeled with fluorescence *in situ* hybridization (FISH) probes (red). Green and red fluorescence images were merged into a single composite image (*Nitrospira* lineage II, yellow; other microorganisms, green).

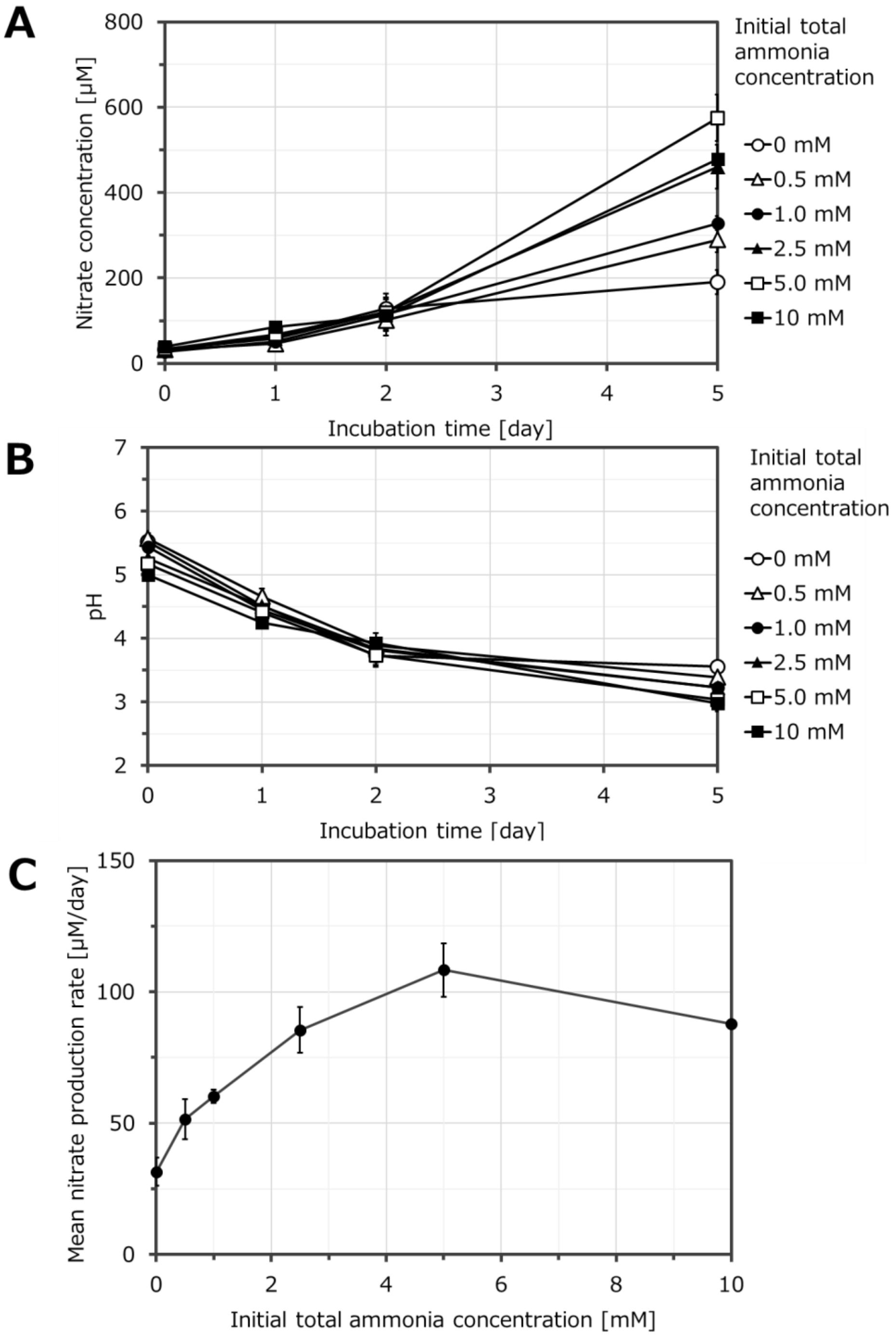

Fig. S8. Test for nitrification rates of AFB enrichment at different ammonia concentrations. Error bars indicate standard deviations of biological triplicates. Changes in **(A)** nitrate concentrations and **(B)** pH of the medium over time at different initial total ammonia concentrations (open circle, 0 mM; open triangle, 0.5 mM; filled circle, 1.0 mM; filled triangle, 2.5 mM; open square, 5.0 mM; filled square, 10 mM). **(C)** Mean nitrate production rates over five days of incubation.

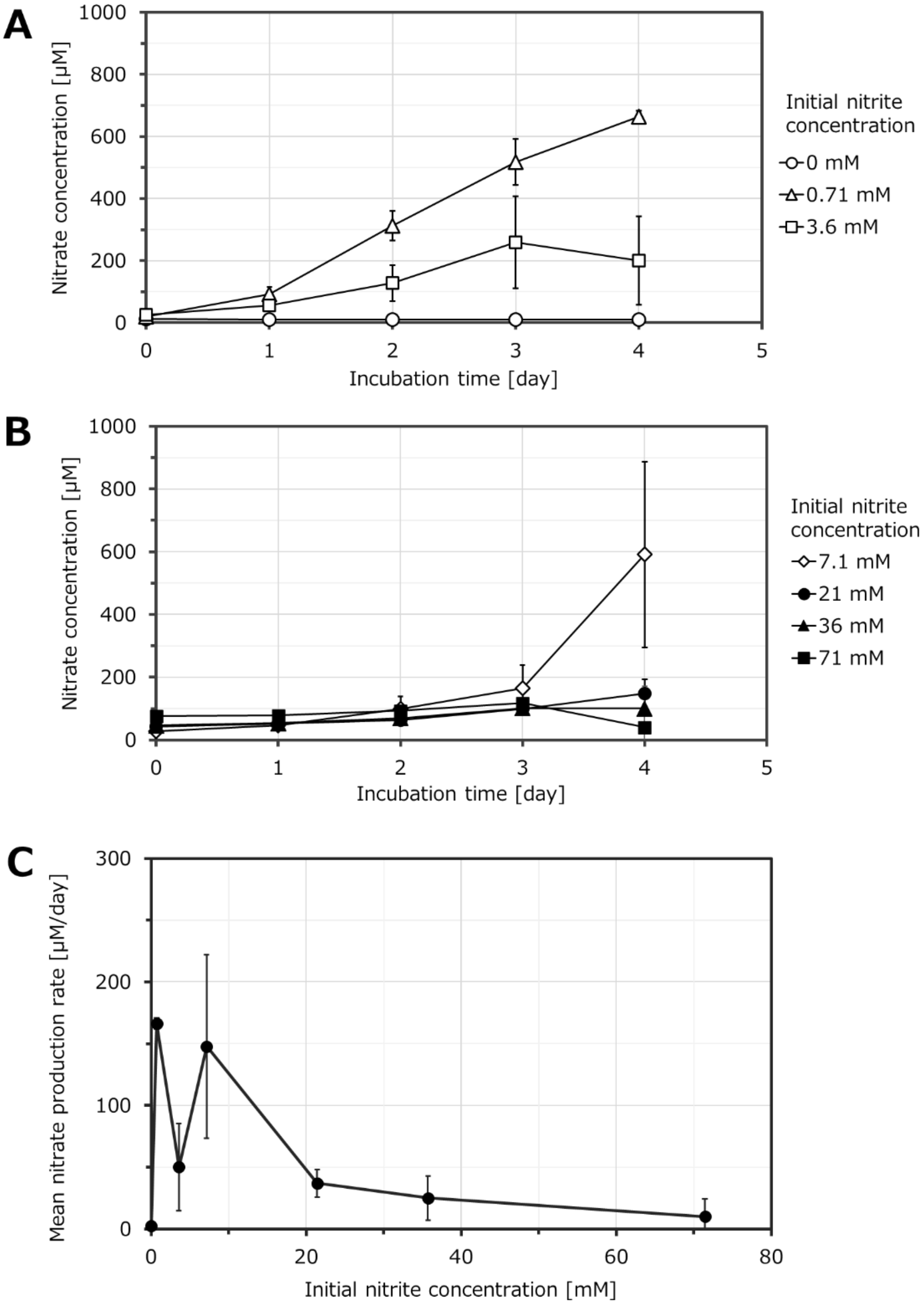

Fig. S9. Test for nitrification rates of NFB enrichment at different nitrite concentrations. Error bars indicate standard deviations of biological triplicates. **(A, B)** Changes in nitrate concentrations over time at different initial nitrite concentrations (open circle, 0 mM; open triangle, 0.71 mM; open square, 3.6 mM; open diamond, 7.1 mM; filled circle, 21 mM; filled triangle, 36 mM; filled square, 71 mM). **(C)** Mean nitrate production rates over four days of incubation.

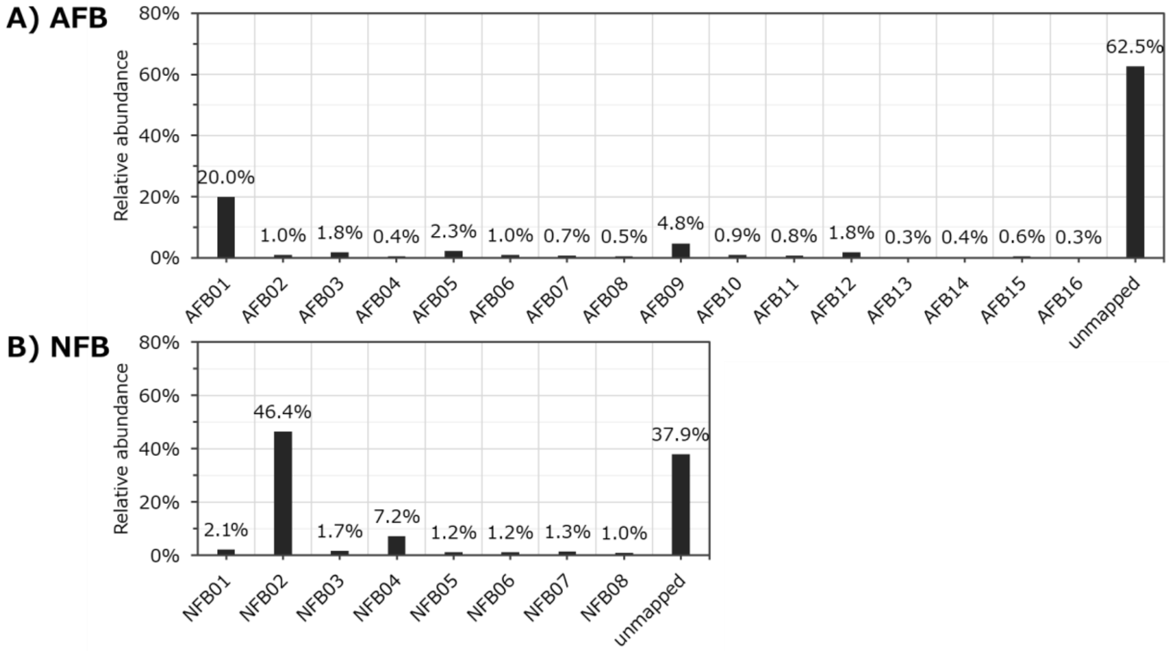

Fig. S10. Relative abundances of MAGs and unmapped reads in **(A)** AFB and **(B)** NFB. The abundances were calculated by mapping HiSeq pair-end short reads to MAGs using CoverM [47].

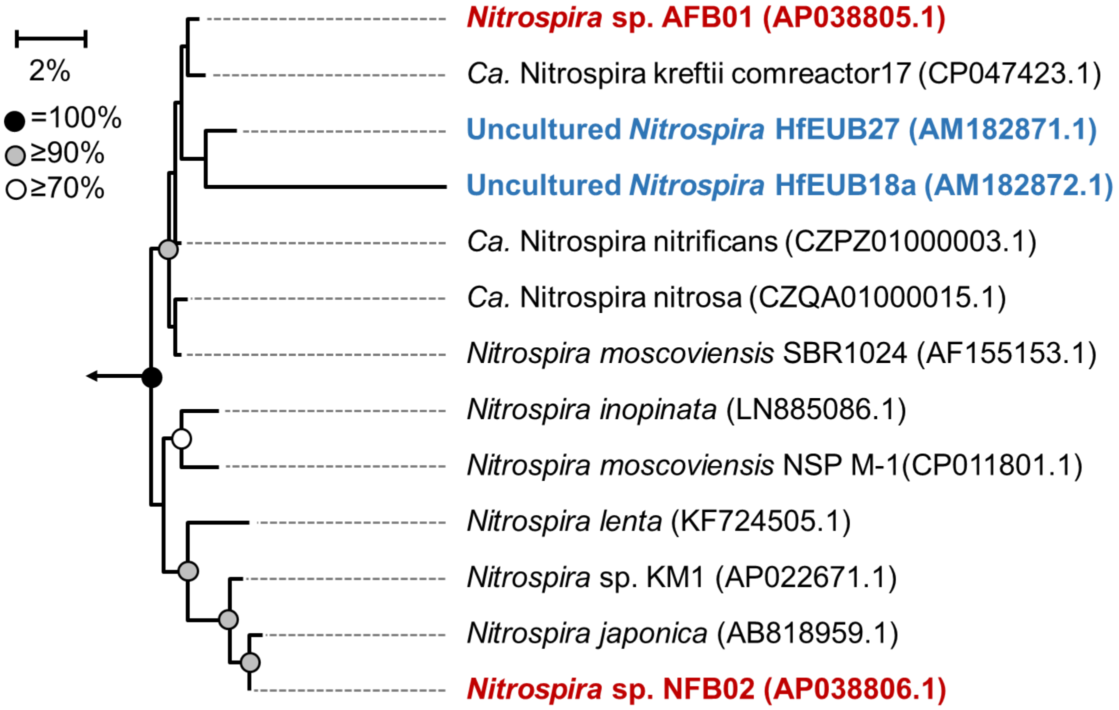

Fig. S11. Phylogenetic analysis of *Nitrospira* lineage II based on the 16S rRNA gene sequences. *Nitrospira* MAGs retrieved in this study are shown as bold red letters. Previously reported clones retrieved from an acidic biofilm reactor [48] are shown as bold blue letters. The scale bar corresponds to 2% estimated sequence divergence. Bootstrap support based on 500 iterations is indicated by colored circles (black, 100%; gray, ≥90%; white, ≥70%). The letters in parentheses indicate accession numbers. The outgroup consisting of *Leptospirillum ferriphilum* strain Fairview (AF356830.1) and *Leptospirillum ferriphilum* voucher BGR:83 (GU168001.1) is indicated by the arrow.

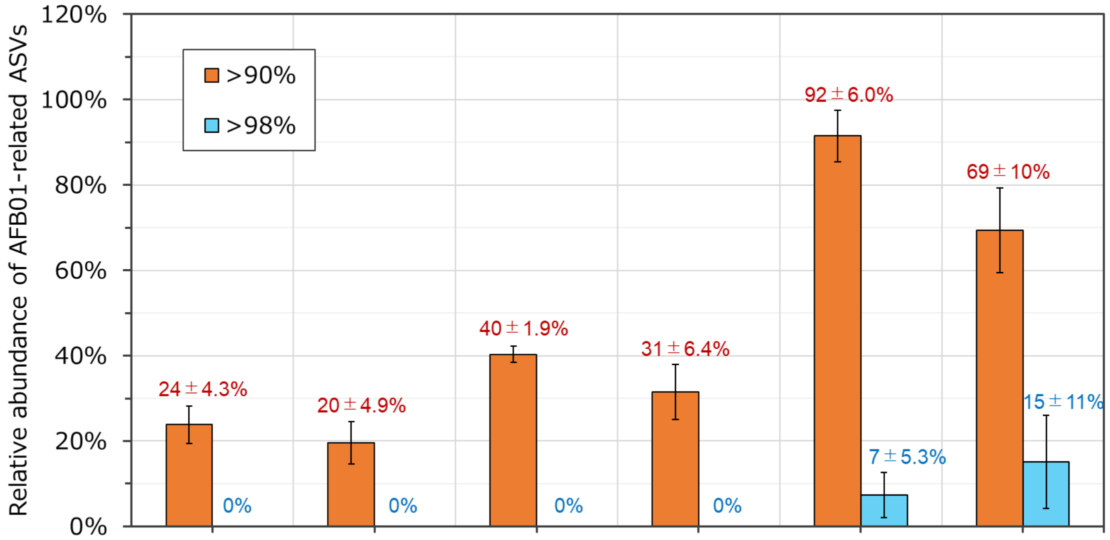

Fertilizer	Chemical	Organic	Chemical	Organic	Chemical	Organic
pH	5.23±0.10	6.95±0.02	6.27±0.06	6.19±0.20	3.35±0.10	3.83±0.14
Field type	Cabbage field		Soybean field		Tea field	

Fig. S12. Relative abundance of AFB01-related ASVs compared to the total ASVs derived from amplicon sequences, targeting comammox *amoA* gene in agricultural soils, obtained in a previous study [10]. ASVs sharing >90% (red) or >98% (blue) nucleotide identity with AFB01 were quantified. Values represent the means ± standard deviations of triplicate soil samples. Bar heights indicate means, and error bars indicate the standard deviations. The table shows the physicochemical properties of the soils, as reported in a previous study [10].

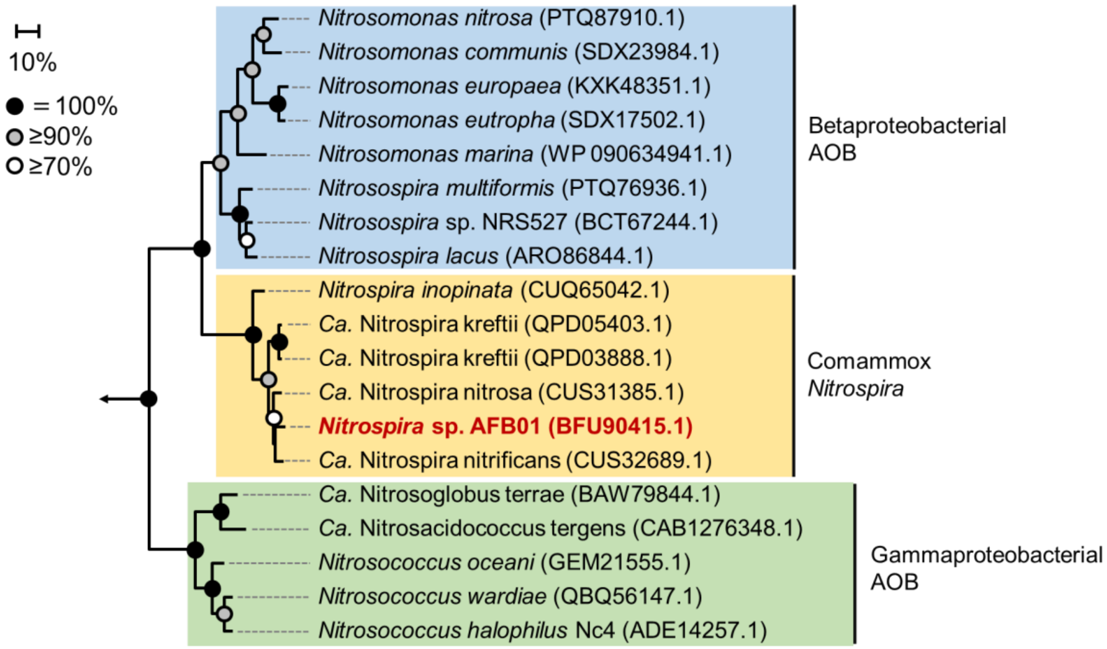

Fig. S13. Phylogeny based on HAO amino-acid sequences of betaproteobacterial AOB (blue box), comammox *Nitrospira* (yellow box), and gammaproteobacterial AOB (green box). *Nitrospira* MAG retrieved in this study is shown as bold red letters. The scale bar corresponds to 10% estimated sequence divergence. Bootstrap support based on 500 iterations is indicated by colored circles (black, 100%; gray, ≥90%; white, ≥70%). The letters in parentheses indicate accession numbers. The outgroup consisting of *Methylomarinovum caldicuralii* (BAN17325.1) and *Methylococcus capsulatus* strain Bath (AAU92745.1) is indicated by the arrow.

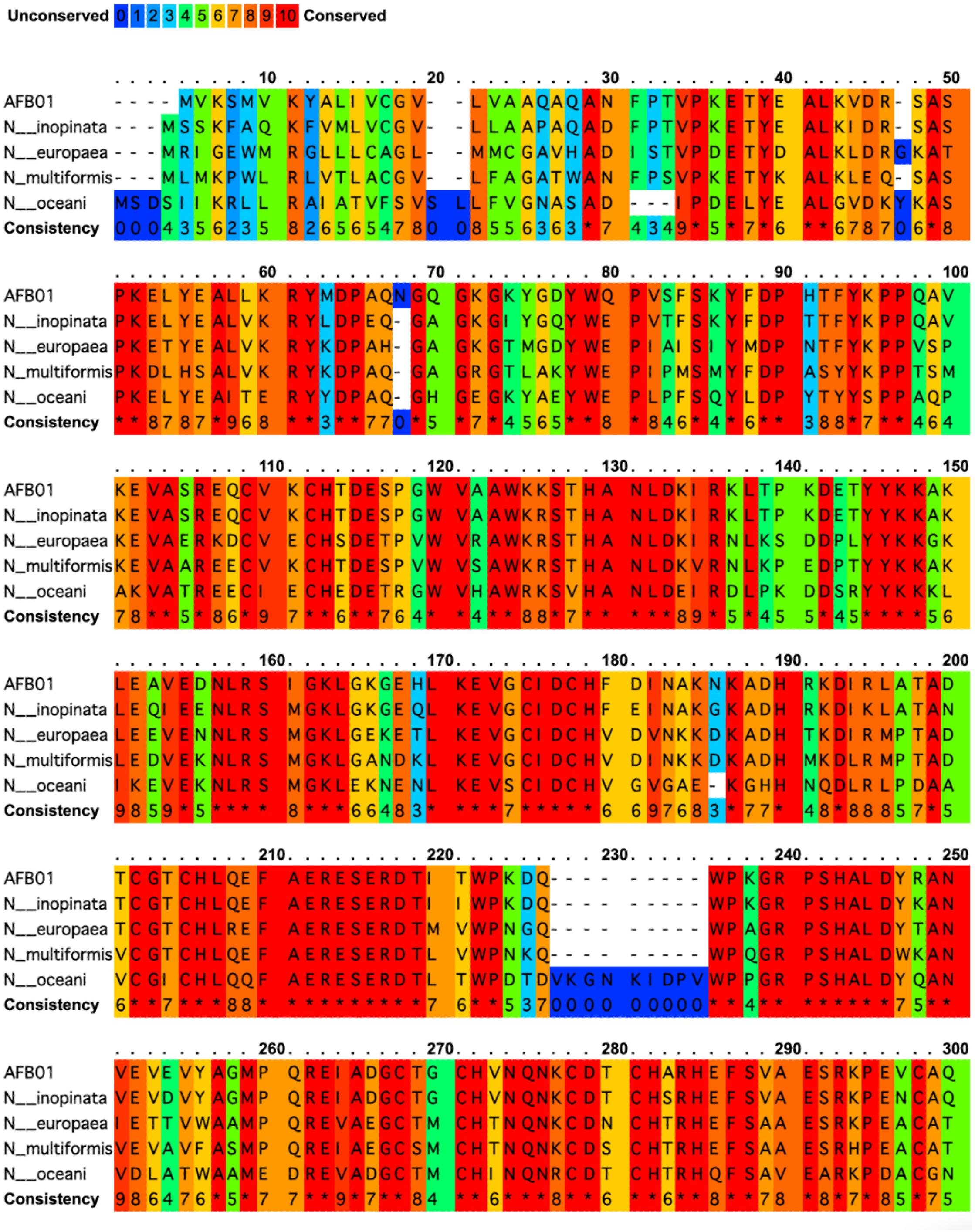

Fig. S14. Continues on next page.

Fig. S14. Continues from the previous page.

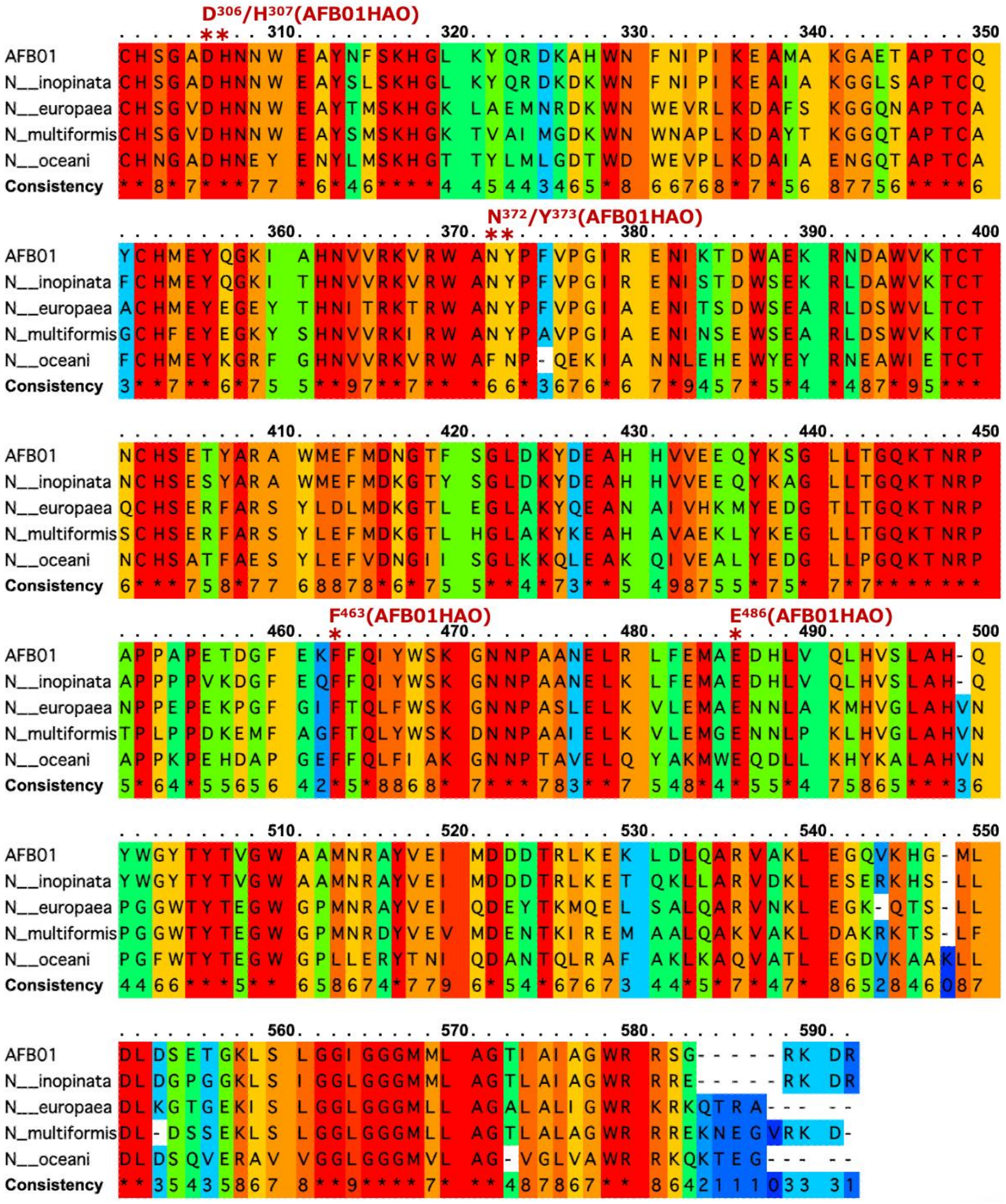

Fig. S14. Multiple alignment of HAOs. HAOs from *Nitrospira* sp. AFB01 (BFU90415.1) reconstructed in this study, *Nitrospira inopinata* (CUQ65042.1), *Nitrosomonas europaea* (KXK48351.1), *Nitrosospira multiformis* (PTQ76936.1), and *Nitrosococcus oceani* (GEM21555.1) were aligned (the letters in parentheses indicate accession numbers). A homology-extended multiple

alignment was conducted using the PRALINE toolbox [49]. The asterisks on top of each alignment block indicate putative active-sites residues referring to *Nitrosomonas europaea* HAO [50]. The color scheme and bottom consistency values show conservation levels according to the color key.

Table S1. The components of medium for cultivation of AFB *Nitrospira* enrichment.

Substance	Concentration [mg/L]
$NaCl$	116
$MgSO_4 \cdot 7H_2O$	40
$CaCl_2 \cdot 2H_2O$	73
KCl	38
$NaHCO_3$	168
$FeCl_2 \cdot 6H_2O$	2
EDTA	4.3
$MnCl_2 \cdot 4H_2O$	0.1
$CoCl_2 \cdot 6H_2O$	0.024
$NiCl_2 \cdot 6H_2O$	0.024
$CuCl_2 \cdot 2H_2O$	0.017
$ZnCl_2$	0.068
$Na_2WO_4 \cdot 2H_2O$	0.033
Na_2MoO_4	0.024
H_3BO_3	0.062

Table S2. The components of medium for cultivation of NFB *Nitrospira* enrichment.

Substance	Concentration [mg/L]
$MgSO_4 \cdot 7H_2O$	61
$CaCl_2 \cdot 2H_2O$	10
$FeSO_4 \cdot 7H_2O$	5
$NaHCO_3$	200
$MnSO_4 \cdot 5H_2O$	0.0542
H_3BO_3	0.0494
$ZnSO_4 \cdot 7H_2O$	0.0431
Na_2MoO_4	0.0276
$CuSO_4 \cdot 5H_2O$	0.0250

Table S3. The number and sum length of reads obtained by metagenomic sequencing.

Sample	Platform	Condition	Number of reads	Sum length [bp]
AFB	Hiseq	Raw	109,098,098	16,473,812,798
		Trimmed	108,717,112	15,813,826,707
		Randomly sampled (66%)[ab]	71,742,450	10,435,591,812
	MinION	Raw	1,647,606	6,797,770,217
		Trimmed	847,599	5,557,350,462
		Length filtered (>7 kbp)[a]	308,230	3,318,989,346
NFB	Hiseq	Raw	66,691,742	10,070,453,042
		Trimmed[b]	66,001,882	9,502,013,971
		Randomly sampled (20%)[a]	13,208,764	1,901,613,538
	MinION	Raw	1,551,633	5,335,484,806
		Trimmed	716,352	4,193,259,119
		Length filtered (>7 kbp)[a]	206,194	2,136,108,238

a) The condition of reads used for hybrid assembly.

b) The condition of reads used to calculate relative abundances of MAGs.

Table S4. The reference genomes used for functional gene annotation.

MAG in this study	Reference genome	Accession*
AFB01	*Candidatus* Nitrospira kreftii	GCA_014058405.1
AFB05	*Nitrobacter hamburgensis* X14	GCA_000013885.1
AFB09	*Nitrosospira multiformis* ATCC 25196	GCA_900169565.1
NFB01	*Nitrospira defluvii*	GCA_000196815.1
	Nitrospira sp. ND1	GCA_900170025.1
NFB02	*Nitrospira japonica*	GCA_900169565.1

*Accession numbers in the NCBI Assembly database [17].

Table S5. The whole genome sequences analyzed to construct the phylogenetic tree of *Nitrospira*.

Genome	Accession*
Nitrospira sp. AFB01	AP038805.1
Nitrospira sp. NFB01	BAAGBG000000000.1
Nitrospira sp. NFB02	AP038806.1
Nitrospira sp. CTRL-LIN-TMP-bin1	GCA_018242685.1
Nitrospira sp. LK70	GCA_011090425.1
Nitrospira sp. RBC093	GCA_902500825.1
Nitrospira sp. LK265	GCA_011090395.1
Nitrospira sp. CR1.1	GCA_014055465.1
Nitrospira sp. MAG_242	GCA_009594945.1
Nitrospira japonica	GCA_900169565.1
Nitrospira moscoviensis NSP M-1	GCA_001273775.1
Nitrospira inopinata	GCA_001458695.1
Candidatus Nitrospira kreftii	GCA_014058405.1
Candidatus Nitrospira nitrificans	GCA_001458775.1
Candidatus Nitrospira nitrosa	GCA_001458735.1
Nitrospira sp. SG-bin1	GCA_002083365.1
Nitrospira lenta	GCA_900403705.1
Nitrospira sp. CR1.3	GCA_014055525.1
Nitrospira sp. CG24D	GCA_002869855.2
Nitrospira sp. RSF13	GCA_005116865.1
Nitrospira defluvii	GCA_000196815.1
Nitrospira sp. ND1	GCA_900170025.1
Nitrospira sp. KM1	GCA_011405515.1
Nitrospira sp. CR1.2	GCA_014055495.1
Nitrospira sp. CG24A	GCA_002869925.2
Nitrospira sp. CG24E	GCA_002869895.2
Nitrospira sp. RSF6	GCA_005116885.1
Leptospirillum ferrooxidans C2-3	GCA_000284315.1
Leptospirillum ferriphilum ML-04	GCA_000299235.1

*Accession numbers in the NCBI Assembly database [17] and Nucleotide database [51].

Table S6. The amino acid sequences analyzed to construct the phylogenetic tree of UT.

Genome	Accession*
Nitrospira sp. AFB01	BFU92727.1
Nitrospira sp. NFB02	BFU96064.1
Nitrospira sp. NFB02	BFU94740.1
Nitrospira sp. CR1.3	MBA5867116.1
Nitrospira japonica	WP_197685400.1
Nitrospira sp. KM1	WP_173050726.1
Nitrospira sp. CTRL-LIN-TMP-bin1	MBS0181153.1
Ca. Nitrosacidococcus tergens	WP_197744830.1
Nitrosococcus oceani	WP_002811941.1
Nitrosococcus watsonii C-113	ADJ27177.1
Nitrosomonas sp. JL21	WP_160827605.1
Nitrosomonas aestuarii	WP_090698662.1
Nitrosomonas oligotropha	WP_160816976.1
Brucella melitensis 16M	AAL51823.1
Brucella abortus 2308	KFJ52486.1
Brucella inopinata	WP_008506104.1
Brucella vulpis	CUW44835.1
Klebsiella pneumoniae CG43	AGX40962.1
Klebsiella pneumoniae subsp. Pneumoniae	AIK82734.1
Yersinia pseudotuberculosis	WP_011192841.1
Yersinia enterocolitica	WP_083163065.1
Desulfovibrio vulgaris str. Hildenborough	3K3F_A
Desulfovibrio aminophilus	WP_027175573.1
Desulfovibrio vulgaris DP4	ABM28909.1
Desulfolutivibrio sulfoxidireducens	QLA18974.1
Desulfovibrio desulfuricans	GEB78918.1
Ca. Nitrososphaera gargensis Ga9.2	AFU57552.1
Ca. Nitrosocosmicus arcticus	WP_144731234.1

*Accession numbers of sequences deposited in the NCBI Protein database [51].

Table S7. The quality of MAGs assessed by CheckM [7].

MAG	Completeness	Contamination	Strain heterogeneity
AFB01	96.76%	3.69%	0%
AFB02	99.21%	0.00%	0%
AFB03	99.20%	0.25%	0%
AFB04	99.06%	0.42%	33.33%
AFB05	98.92%	1.95%	50.00%
AFB06	98.66%	1.52%	66.67%
AFB07	98.64%	1.43%	50.00%
AFB08	98.07%	0.47%	0%
AFB09	96.87%	0.48%	0%
AFB10	96.83%	1.81%	0%
AFB11	96.52%	0.43%	0%
AFB12	95.50%	0.76%	33.33%
AFB13	91.98%	0.93%	0%
AFB14	91.55%	2.70%	25.00%
AFB15	91.48%	0.93%	0%
AFB16	90.63%	2.97%	0%
NFB01	97.68%	2.93%	71.43%
NFB02	95.85%	2.73%	0%
NFB03	98.29%	6.41%	0%
NFB04	96.70%	3.30%	0%
NFB05	94.92%	1.46%	42.86%
NFB06	92.97%	2.14%	0%
NFB07	90.68%	5.06%	25.00%
NFB08	90.61%	3.82%	28.57%

Table S8. The sequencing statistics of high-quality MAGs reconstructed in this study.

MAG	Number of sequences	Sum length [bp]	Minimum length [bp]	Average length [bp]	Maximum length [bp]	N50 [bp]
AFB01	1	4,565,847	4,565,847	4,565,847	4,565,847	4,565,847
AFB02	12	4,199,579	14,688	349,965	1,156,403	841,454
AFB03	9	3,655,497	146,555	406,166	1,034,553	462,961
AFB04	44	5,339,575	3,814	121,354	508,137	193,188
AFB05	5	3,628,915	6,601	725,783	3,594,853	3,594,853
AFB06	27	3,390,888	3,146	125,588	659,593	492,776
AFB07	46	3,455,467	3,737	75,119	268,525	145,792
AFB08	16	4,275,557	35,893	267,222	525,752	434,401
AFB09	4	3,140,534	228,701	785,134	1,270,034	1,211,744
AFB10	17	2,443,641	21,287	143,744	277,437	225,111
AFB11	51	5,585,764	2,614	109,525	541,639	189,760
AFB12	44	3,145,856	2,716	71,497	559,042	167,464
AFB13	88	2,498,585	2,599	28,393	117,866	58,268
AFB14	72	2,783,573	2,672	38,661	147,224	50,237
AFB15	8	2,556,469	9,864	319,559	800,093	623,934
AFB16	80	3,129,541	2,584	39,119	255,585	60,628
NFB01	22	4,143,616	3,655	188,346	530,946	400,767
NFB02	1	4,071,157	4,071,157	4,071,157	4,071,157	4,071,157
NFB03	10	6,315,493	100,313	631,549	2,093,246	988,496
NFB04	5	3,535,198	277,957	707,040	1,168,372	1,041,216
NFB05	55	3,375,947	2,653	61,381	365,332	100,254
NFB06	34	6,126,181	2,694	180,182	849,801	412,665
NFB07	151	4,801,903	2,518	31,801	151,796	58,357
NFB08	50	8,203,680	3,777	164,074	1,304,736	492,466

Table S9. The read coverage of high-quality MAGs reconstructed in this study.

MAG	Short read coverage			Long read coverage		
	Range	Median	Average	Range	Median	Average
AFB01	485.7	485.70	485.70	194.73	194.73	194.73
AFB02	26.11−55.06	27.34	29.87	3.51−18.36	7.65	8.07
AFB03	45.65−52.63	48.97	49.27	11.61−13.58	12.55	12.58
AFB04	10.31−13.47	11.93	11.92	1.11−7.18	4.11	3.97
AFB05	15.29−51.25	17.89	24.87	7.98−22.4	11.90	13.29
AFB06	23.03−29.81	26.11	25.98	0.38−7.28	5.58	4.63
AFB07	7.24−47.29	18.53	19.34	3.64−24.69	12.38	12.02
AFB08	12.68−16.03	14.88	14.72	3.98−6.59	5.31	5.31
AFB09	113.29−121.71	119.38	118.44	27.11−29.87	28.41	28.45
AFB10	22.7−26.85	23.85	24.28	3.99−7.99	6.40	6.36
AFB11	17.45−26.7	20.76	20.90	0.29−9.24	6.18	5.87
AFB12	41.41−51.6	46.75	47.07	10.22−20.07	14.40	14.95
AFB13	6.88−11.29	8.40	8.46	0−5.19	2.35	2.14
AFB14	7.33−14.09	8.85	9.01	0.34−13.66	2.13	2.31
AFB15	15.13−16.4	15.49	15.61	1.13−5.67	4.10	3.93
AFB16	2.59−10.25	8.43	8.11	0−6.05	2.67	2.75
NFB01	5.74−14.52	10.58	10.51	11.38−25.5	16.43	16.55
NFB02	199.94	199.94	199.94	244.43	244.43	244.43
NFB03	7.89−8.43	8.16	8.18	12.26−15.26	13.63	13.56
NFB04	34.57−37.1	35.48	35.72	25.36−29.92	29.69	28.68
NFB05	2.75−8.1	5.41	5.41	0.39−7.87	5.18	4.49
NFB06	2.53−23.93	5.83	6.55	2.23−19.72	8.06	8.49
NFB07	4.11−18.17	6.43	6.42	0.6−38.93	5.49	5.81
NFB08	1.84−8.6	4.73	4.40	4.29−14.36	8.93	8.66

Table S10. The sequences homologous to genes or proteins of MAGs reconstructed in this study.

MAG	Query gene/protein	Scientific Name	Query Cover	E value	Per. ident	Accession*
AFB01	16S rRNA gene	*Ca.* Nitrospira kreftii	100%	0	98.96%	CP047423.1
AFB02	16S rRNA gene	*Gordonia* sp.	97%	0	97.85%	MH699192.1
AFB03	16S rRNA gene	*Micropepsis pineolensis*	97%	0	91.82%	NR_156848.1
AFB04	16S rRNA gene	*Gordonia bronchialis*	100%	0	99.93%	CP046320.1
AFB05	16S rRNA gene	*Nitrobacter winogradskyi*	100%	0	99.33%	NR_074324.1
AFB06	16S rRNA gene	*Sphingopyxis terrae* subsp.	100%	0	99.93%	CP013342.1
AFB07	16S rRNA gene	*Rhodoferax sediminis*	100%	0	98.56%	CP035503.1
AFB08	RpoB	*Rhodoplanes roseus*	97%	0	86.5%	WP_111418986.1
AFB09	16S rRNA gene	*Nitrosospira lacus*	100%	0	98.89%	CP021106.3
AFB10	16S rRNA gene	*Ectothiorhodosinus mongolicus*	99%	0	90.12%	CP023018.1
AFB11	16S rRNA gene	*Reyranella* sp.	97%	0	98.01%	KY176929.1
AFB12	16S rRNA gene	*Rhodoferax sediminis*	100%	0	98.24%	CP035503.1
AFB13	16S rRNA gene	*Desulfofundulus solfataricus*	98%	0	82.59%	NR_036846.1
AFB14	16S rRNA gene	*Limisphaera ngatamarikiensis*	90%	0	87.56%	NR_134756.1
AFB15	16S rRNA gene	*Fimbriimonas ginsengisoli*	100%	0	91.50%	CP007139.1
AFB16	16S rRNA gene	*Tepidiforma bonchosmolovskayae*	99%	0	94.63%	CP042829.1
NFB01	16S rRNA gene	*Nitrospira defluvii*	100%	0	99.09%	FP929003.1
NFB02	16S rRNA gene	*Nitrospira japonica*	100%	0	99.61%	LT828648.1
NFB03	16S rRNA gene	*Ilumatobacter coccineus*	100%	0	91.04%	AP012057.1
NFB04	16S rRNA gene	*Gemmatirosa kalamazoonesis*	99%	0	87.82%	CP007128.1
NFB05	16S rRNA gene	*Ca.* Viadribacter manganicus	100%	0	96.99%	CP013244.1
NFB06	RpoB	*Aquihabitans* sp. G128	99%	0	79.39%	WP_216997201.1
NFB07	RpoA	*Piscinibacter* sp.	100%	0	95.17%	MBP6778588.1
NFB08	16S rRNA gene	*Caldilinea aerophila*	99%	0	82.90%	NR_074397.1

*Accession numbers of sequences deposited in the NCBI Nucleotide and Protein database [51].

Table S11. Genomic features of *Nitrospira* MAGs reconstructed in this study.

Characteristics	AFB01	NFB01	NFB02
Gap ratio	0%	0%	0%
GC content	55.5%	58.4%	59.3%
Number of CDSs	4,465	3,918	3,896
Coding ratio	85.6%	86.6%	87.5%
Number of rRNAs	3	3	3
Number of tRNAs	47	48	46
Number of CRISPRs	1	1	1

Table S12. Mash distance, ANI (average nucleotide identity), and AAI (average amino-acid identity) between *Nitrospira* MAGs in this study and the five closest genomes.

Query genome	Subject genome	Accession*	Mash distance	ANI	AAI
AFB01	*Nitrospira* sp. CTRL-LIN-TMP-bin1	GCA_018242685.1	0.044	94.8%	95.0%
AFB01	*Nitrospira* sp. LK70	GCA_011090425.1	0.130	85.2%	85.9%
AFB01	*Nitrospira* sp. ACE_NIT13	GCA_019636835.1	0.130	85.8%	87.1%
AFB01	*Nitrospira* sp. RBC093	GCA_902500825.1	0.130	85.4%	87.2%
AFB01	*Nitrospira* sp. LK265	GCA_011090395.1	0.157	84.7%	85.9%
NFB02	*Nitrospira japonica*	GCA_900169565.1	0.144	84.0%	87.4%
NFB02	*Nitrospira moscoviensis* NSP M-1	GCA_001273775.1	0.220	74.1%	72.1%
NFB02	*Nitrospira* sp. LK265	GCA_011090395.1	0.220	73.4%	70.3%
NFB02	*Nitrospira* sp. LK70	GCA_011090425.1	0.220	72.7%	70.0%
NFB02	*N.* sp. K_Offshore_80m_m2_040	GCA_016873435.1	0.220	72.5%	66.8%

*Accession numbers in the NCBI Assembly database [17].

Table S13. Distribution pattern of metabolic pathways in *Nitrospira* MAGs retrieved in this study.

Metabolism	Pathway/Protein	AFB01	NFB02
Nitrification	Ammonia monooxygenase (AmoABC)	black	white
	Hydroxylamine:ubiquinone reduction module (HURM)	black	white
	Nitrite oxidoreductase (NXR)	black	black
	Copper-containing nitrite reductase (NirK)	black	black
	Assimilatory nitrite reductase (NirA)	white	white
	Octaheme cytochrome *c* (OCC) nitrite reductase	white	black
	Transmembrane Rieske/cytochrome *b* complex	black	black
Nitrogen assimilation	Cyanate hydratase	black	black
	Amt-type ammonium transporter	white	black
	Rh-type ammonium transporter	black	white
Urea degradation	Urease enzyme (UreABC)	black	black
	Urease accessory proteins (UreDEFG)	gray	black
Urea transport	ABC-type Urea transporter (UrtABCDE)	black	black
	Urea transporter (*Nitrospira* UT-1)	white	black
	Urea transporter (*Nitrospira* UT-2)	black	black
Energy metabolism	NADH:quinone oxidoreductase (complex I)	black	black
	Succinate dehydrogenase/fumarate reductase (complex II)	black	black
	Cytochrome bc_1 complex (complex III)	black	gray
	Cytochrome *bd* terminal oxidase (complex IV)	black	black
	F-type ATPase (complex V)	black	black
Carbon metabolism	Oxidative TCA cycle	black	black
	Reductive TCA cycle	gray	gray
	Pentose phosphate pathway	black	black
	Glycolysis (Embden-Meyerhof pathway)	black	black
	Gluconeogenesis	black	gray
	Pyruvate oxidation	black	black
Formate metabolism	Formate transporter	white	black
	NAD^+-dependent soluble formate dehydrogenase (FdsABG)	white	black
	Molybdenum-dependent formate dehydrogenase (FdhF)	black	white
	Accessory sulfurtransferase (FdhD)	black	white
Hydrogen metabolism	Group 3b [NiFe]-hydrogenase (HYD)	black	white
	Hydrogenase maturation	gray	white
Detoxification of superoxide	Manganese and iron superoxide dismutase (Fe/MnSOD)	black	black
	Copper and zinc superoxide dismutase (Cu/ZnSOD)	white	black
	Heme catalase/peroxidase HPI (KatG)	white	white
	Hydroperoxidase II HPII (KatA)	black	white
Cation/H^+ antiport	Multiple resistance and pH adaptation (Mrp) antiporter	black	black

The colors indicate the completeness of pathways (black, complete; gray, incomplete; white, all the genes involved in the pathway are absent).

Table S14. Homologs of acid tolerance-related proteins.

Protein	Query species	Query accession[a]	Subject locus tag[b]		Subject accession number[ab]	
			AFB01	NFB02	*Ca.* N. kreftii	*N. japonica*
Cfa	*Escherichia coli*	AIZ91234.1	AFB01_17680	NFB02_21140	QPD04045.1	SLM47863.1
BetL	*Listeria monocytogenes*	CAD00170.1	NA	NA	NA	NA
GshA	*Escherichia coli*	AIZ90267.1	NA	NA	NA	NA
GshB	*Escherichia coli*	AIZ90024.1	NA	NA	NA	NA
TrePP	*Lactococcus lactis*	SPS12283.1	AFB01_17880	NA	NA	NA
PgmB	*Lactococcus lactis*	AWN65065.1	AFB01_17890	NFB02_30420	QPD05054.1	SLM48800.1
OtsB	*Propionibacterium freudenreichii*	CEH09822.1	AFB01_07100	NFB02_16040	QPD02920.1	SLM47290.1
HdcA	*Streptococcus thermophilus*	CBX24604.1	NA	NA	NA	NA
HdcP	*Streptococcus thermophilus*	CBK46770.1	NA	NA	NA	NA
HdcB	*Streptococcus thermophilus*	CBK46771.1	NA	NA	NA	NA
YbaS	*Escherichia coli*	ANK05841.1	NA	NA	NA	NA
GadC	*Escherichia coli*	CAD6014273.1	NA	NA	QPD06342.1	NA
MurG	*Lactococcus lactis*	XUT99774.1	AFB01_37630	NFB02_21580	QPD05618.1	SLM47905.1
HdeA	*Escherichia coli*	CAD5999836.1	NA	NA	NA	NA
HdeB	*Escherichia coli*	CAD5999843.1	NA	NA	NA	NA
Hsp31	*Escherichia coli*	CUU94115.1	NA	NA	NA	NA
AtpA	*Propionibacterium acidipropionici*	AFV89736.1	AFB01_05690	NFB02_18170	QPD06020.1	SLM47590.1
CA	*Helicobacter pylori*	WP_431341301.1	AFB01_04060	NFB02_29980	QPD06192.1	NA

[a]Accession numbers of sequences deposited in the NCBI Protein database [51].

[b]'NA' indicates that homologous proteins are absent in the subject genomes.

Supplementary datasets (separate file)

Dataset S1. Genes involved in respiratory chain, TCA cycles, pentose phosphate pathway, glycolysis, gluconeogenesis, pyruvate oxidation, nitrification, nitrogen assimilation, detoxification of superoxide, formate metabolism, hydrogen metabolism, and Mrp antiporter in *Nitrospira* MAGs retrieved in this study. 'NA' in the column 'Gene/protein abbreviation' indicate that the products have no abbreviations.

Dataset S2. Proteins present in *Nitrospira* sp. AFB01 but absent in *Candidatus* Nitrospira kreftii, *Candidatus* Nitrospira nitrificans, *Candidatus* Nitrospira nitrosa, and *Nitrospira inopinata*. Comparative genomics was performed using OrthoVenn2 [18].

Dataset S3. Proteins present in *Nitrospira* sp. NFB02 but absent in *Nitrospira japonica* NJ1, *Nitrospira* sp. KM1, and *Nitrospira moscoviensis* NSP M-1. Comparative genomics was performed using OrthoVenn2 [18].

SI References

1. Takahashi Y, Fujitani H, Hirono Y, Tago K, Wang Y, Hayatsu M, *et al*. Enrichment of Comammox and Nitrite-Oxidizing *Nitrospira* From Acidic Soils. *Front Microbiol.* 2020;**11**:1737. https://doi.org/10.3389/fmicb.2020.01737
2. Kajitani R, Toshimoto K, Noguchi H, Toyoda A, Ogura Y, Okuno M, *et al*. Efficient de novo assembly of highly heterozygous genomes from whole-genome shotgun short reads. *Genome Res*. 2014;**24**(8):1384–95. https://doi.org/10.1101/gr.170720.113
3. Shen W, Le S, Li Y, Hu F. SeqKit: A Cross-Platform and Ultrafast Toolkit for FASTA/Q File Manipulation. *PLOS ONE*. 2016;**11**(10):e0163962. https://doi.org/10.1371/journal.pone.0163962
4. De Coster W, D'Hert S, Schultz DT, Cruts M, Van Broeckhoven C. NanoPack: visualizing and processing long-read sequencing data. *Bioinformatics*. 2018;**34**(15):2666–9. https://doi.org/10.1093/bioinformatics/bty149
5. Bertrand D, Shaw J, Kalathiyappan M, Ng AHQ, Kumar MS, Li C, *et al.* Hybrid metagenomic

assembly enables high-resolution analysis of resistance determinants and mobile elements in human microbiomes. *Nat Biotechnol*. 2019;**37**:937–44. https://doi.org/10.1038/s41587-019-0191-2

6. Kang DD, Li F, Kirton E, Thomas A, Egan R, An H, *et al*. MetaBAT 2: an adaptive binning algorithm for robust and efficient genome reconstruction from metagenome assemblies. *PeerJ*. 2019;**7**:e7359. https://doi.org/10.7717/peerj.7359
7. Parks DH, Imelfort M, Skennerton CT, Hugenholtz P, Tyson GW. CheckM: assessing the quality of microbial genomes recovered from isolates, single cells, and metagenomes. *Genome Res*. 2015;**25**(7):1043–55. https://doi.org/10.1101/gr.186072.114
8. Sakoula D, Koch H, Frank J, Jetten MSM, van Kessel M, Lücker S. Enrichment and physiological characterization of a novel comammox *Nitrospira* indicates ammonium inhibition of complete nitrification. *ISME J*. 2021;**15**:1010–24. https://doi.org/10.1038/s41396-020-00827-4
9. Aroney STN, Newell RJP, Nissen J, Camargo AP, Tyson GW, Woodcroft BJ. CoverM: Read coverage calculator for metagenomics (v0.7.0). *Zenodo*. 2024. https://doi.org/10.5281/zenodo.10531254
10. Ohbayashi T, Wang Y, Aoyagi LN, Hara S, Tago K, Hayatsu M. Diversity of the Hydroxylamine Oxidoreductase (HAO) Gene and Its Enzyme Active Site in Agricultural Field Soils. *Microbes Environ*. 2023;**38**(4). https://doi.org/10.1264/jsme2.ME23068
11. Callahan BJ, McMurdie, PJ., Rosen, MK, Han AW, Johnson AJA, Holmes SP. DADA2: High-resolution sample inference from Illumina amplicon data. *Nat Methods*. 2016;**13**:581–3. https://doi.org/10.1038/nmeth.3869
12. Morgan M, Anders S, Lawrence M, Aboyoun P, Pagès H, Gentleman R. ShortRead: a bioconductor package for input, quality assessment and exploration of high-throughput sequence data, *Bioinformatics*. 2009;**25**(19): 2607–8. https://doi.org/10.1093/bioinformatics/btp450
13. Pagès H, Aboyoun P, Gentleman R, DebRoy S. Biostrings: Efficient manipulation of biological strings. 2025. https://bioconductor.org/packages/Biostrings
14. R Core Team. R: A language and environment for statistical computing. 2021. https://www.R-

project.org/

15. Martin M. Cutadapt removes adapter sequences from high-throughput sequencing reads. *EMBnet J*. 2011;**17**:10–2. https://doi.org/10.14806/ej.17.1.200
16. Camacho C, Coulouris G, Avagyan V. Ma N, Papadopoulos J, Bealer K *et al*. BLAST+: architecture and applications. *BMC Bioinformatics*. 2009;**10**:421. https://doi.org/10.1186/1471-2105-10-421
17. Kitts PA, Church DM, Thibaud-Nissen F, Choi J, Hem V, Sapojnikov V, *et al*. Assembly: a resource for assembled genomes at NCBI. *Nucleic Acids Res*. 2016;**44**(D1):D73–80. https://doi.org/10.1093/nar/gkv1226
18. Xu L, Dong Z, Fang L, Luo Y, Wei Z, Guo H, *et al*. OrthoVenn2: a web server for whole-genome comparison and annotation of orthologous clusters across multiple species. *Nucleic Acids Res*. 2019;**47**(W1):W52–8. https://doi.org/10.1093/nar/gkz333
19. Koch H, van Kessel M, Lücker S. Complete nitrification: insights into the ecophysiology of comammox *Nitrospira*. *Appl Microbiol Biotechnol*. 2019;**103**(1):177–89. https://doi.org/10.1007/s00253-018-9486-3
20. Caranto JD, Lancaster KM. Nitric oxide is an obligate bacterial nitrification intermediate produced by hydroxylamine oxidoreductase. *Proc Natl Acad Sci USA*. 2017;**114**(31):8217–22. https://doi.org/10.1073/pnas.1704504114
21. Lehtovirta-Morley LE. Ammonia oxidation: Ecology, physiology, biochemistry and why they must all come together. *FEMS Microbiol Lett*. 2018;**365**(9). https://doi.org/10.1093/femsle/fny058
22. Daims H, Lücker S, Wagner M. A New Perspective on Microbes Formerly Known as Nitrite-Oxidizing Bacteria. *Trends Microbiol*. 2016;**24**(9):699–712. https://doi.org/10.1016/j.tim.2016.05.004
23. Koch H, Lücker S, Albertsen M, Kitzinger K, Herbold C, Spieck E, *et al*. Expanded metabolic versatility of ubiquitous nitrite-oxidizing bacteria from the genus *Nitrospira*. *Proc Natl Acad Sci USA*. 2015;**112**(36):11371–6. https://doi.org/10.1073/pnas.1506533112

24. Ushiki N, Fujitani H, Shimada Y, Morohoshi T, Sekiguchi Y, Tsuneda S. Genomic Analysis of Two Phylogenetically Distinct *Nitrospira* Species Reveals Their Genomic Plasticity and Functional Diversity. *Front Microbiol.* 2017;**8**:2637. https://doi.org/10.3389/fmicb.2017.02637

25. Daims H, Lebedeva EV, Pjevac P, Han P, Herbold C, Albertsen M, *et al*. Complete nitrification by *Nitrospira* bacteria. *Nature*. 2015;**528**(7583):504–9. https://doi.org/10.1038/nature16461

26. Palatinszky M, Herbold C, Jehmlich N, Pogoda M, Han P, von Bergen M, *et al*. Cyanate as an energy source for nitrifiers. *Nature*. 2015;**524**(7563):105–8. https://doi.org/10.1038/nature14856

27. Yang Y, Daims H, Liu Y, Herbold CW, Pjevac P, Lin JG, *et al*. Activity and Metabolic Versatility of Complete Ammonia Oxidizers in Full-Scale Wastewater Treatment Systems. *mBio*. 2020;**11**(2). https://doi.org/10.1128/mbio.03175-19

28. Spasov E, Tsuji JM, Hug LA, Doxey AC, Sauder LA, Parker WJ, *et al*. High functional diversity among *Nitrospira* populations that dominate rotating biological contactor microbial communities in a municipal wastewater treatment plant. *ISME J*. 2020;**14**(7):1857–72. https://doi.org/10.1038/s41396-020-0650-2

29. Palomo A, Pedersen AG, Fowler SJ, Dechesne A, Sicheritz-Pontén T, Smets BF. Comparative genomics sheds light on niche differentiation and the evolutionary history of comammox *Nitrospira*. *ISME J*. 2018;**12**(7):1779–93. https://doi.org/10.1038/s41396-018-0083-3

30. Koch H, Galushko A, Albertsen M, Schintlmeister A, Gruber-Dorninger C, Lücker S, *et al.* Growth of nitrite-oxidizing bacteria by aerobic hydrogen oxidation. *Science.* 2014;**345**(6200):1052–4. https://doi.org/10.1126/science.1256985

31. Johnson LA, Hug LA. Distribution of reactive oxygen species defense mechanisms across domain bacteria. *Free Radic Biol Med.* 2019;**140**:93–102. https://doi.org/10.1016/j.freeradbiomed.2019.03.032

32. Lücker S, Wagner M, Maixner F, Pelletier E, Koch H, Vacherie B, *et al.* A *Nitrospira* metagenome illuminates the physiology and evolution of globally important nitrite-oxidizing bacteria. *Proc Natl Acad Sci USA*. 2010;**107**(30):13479–84. https://doi.org/10.1073/pnas.1003860107

33. Putnoky P, Kereszt A, Nakamura T, Endre G, Grosskopf E, Kiss P, *et al*. The *pha* gene cluster of

Rhizobium meliloti involved in pH adaptation and symbiosis encodes a novel type of K^+ efflux system. *Mol Microbiol*. 1998;**28**(6):1091–101. https://doi.org/10.1046/j.1365-2958.1998.00868.x

34. Ito M, Guffanti AA, Oudega B, Krulwich TA. *mrp*, a multigene, multifunctional locus in *Bacillus subtilis* with roles in resistance to cholate and to Na^+ and in pH homeostasis. *J Bacteriol.* 1999;**181**(8):2394–402. https://doi.org/10.1128/jb.181.8.2394-2402.1999
35. Kajiyama Y, Otagiri M, Sekiguchi J, Kosono S, Kudo T. Complex formation by the *mrpABCDEFG* gene products, which constitute a principal Na^+/H^+ antiporter in *Bacillus subtilis. J Bacteriol*. 2007;**189**(20):7511–4. https://doi.org/10.1128/jb.00968-07
36. Swartz TH, Ikewada S, Ishikawa O, Ito M, Krulwich TA. The Mrp system: a giant among monovalent cation/proton antiporters? *Extremophiles*. 2005;**9**(5):345–54. https://doi.org/10.1007/s00792-005-0451-6
37. Chang YY, Cronan JE. Membrane cyclopropane fatty acid content is a major factor in acid resistance of *Escherichia coli*. *Mol Microbiol*. 1999;**33**:249–59. https://doi.org/10.1046/j.1365-2958.1999.01456.x
38. Sheehan VM, Sleator RD, Hill C, Fitzgerald GF. Improving gastric transit, gastrointestinal persistence and therapeutic efficacy of the probiotic strain *Bifidobacterium breve* UCC2003. *Microbiology*. 2007;**153**(10):3563–71. https://doi.org/10.1099/mic.0.2007/006510-0
39. Zhang J, Fu RY, Hugenholtz J, Li Y, Chen J. Glutathione Protects *Lactococcus lactis* against Acid Stress. *Appl Environ Microbiol*. 2007;**73**(16). https://doi.org/10.1128/AEM.02787-06
40. Carvalho AL, Cardoso FS, Bohn A, Neves AR, Santos H. Engineering Trehalose Synthesis in *Lactococcus lactis* for Improved Stress Tolerance. *Appl Environ Microbiol*. 2011;**77**(12). https://doi.org/10.1128/AEM.02922-10
41. Trip H, Mulder NL, Lolkema JS. Improved Acid Stress Survival of *Lactococcus lactis* Expressing the Histidine Decarboxylation Pathway of *Streptococcus thermophilus* CHCC1524. *J Biol Chem*. 2012;**287**(14):11195–204. https://doi.org/10.1074/jbc.M111.330704
42. Lu P, Ma D, Chen Y, Guo Y, Chen GQ, Deng H, *et al*. L-glutamine provides acid resistance for *Escherichia coli* through enzymatic release of ammonia. *Cell Res*. 2013;**23**:635–44.

https://doi.org/10.1038/cr.2013.13

43. Zhang J, Caiyin, Q, Feng, W, Zhao X, Qiao B, Guangrong Z, *et al*. Enhance nisin yield via improving acid-tolerant capability of *Lactococcus lactis* F44. *Sci Rep*. 2016;**6**:27973. https://doi.org/10.1038/srep27973
44. Xu Y, Zhao Z, Tong W, Ding Y, Liu B, Shi Y, *et al*. An acid-tolerance response system protecting exponentially growing *Escherichia coli*. *Nat Commun*. 2020;**11**:1496. https://doi.org/10.1038/s41467-020-15350-5
45. Guan N, Shin H, Chen RR, Li J, Liu L, Du G, *et al*. Understanding of how *Propionibacterium acidipropionici* respond to propionic acid stress at the level of proteomics. *Sci Rep*. 2014;**4**:6951. https://doi.org/10.1038/srep06951
46. Campestre C, Luca VD, Carradori S, Grande R, Carginale V, Scaloni A, *et al*. Carbonic Anhydrases: New Perspectives on Protein Functional Role and Inhibition in *Helicobacter pylori*. *Front Microbiol*. 2021;**12**:629163. https://doi.org/10.3389/fmicb.2021.629163
47. Aroney STN, Newell RJP, Nissen J, Camargo AP, Tyson GW, Woodcroft BJ. CoverM: Read coverage calculator for metagenomics (v0.7.0). *Zenodo*. 2024. https://doi.org/10.5281/zenodo.10531254
48. Gieseke A, Tarre S, Green M, de Beer D. Nitrification in a biofilm at low pH values: role of *in situ* microenvironments and acid tolerance. *Appl Environ Microbiol.* 2006;**72**(6):4283–92. https://doi.org/10.1128/AEM.00241-06
49. Simossis VA, Heringa J. PRALINE: a multiple sequence alignment toolbox that integrates homology-extended and secondary structure information. *Nucleic Acids Res*. 2005;**33**:W289–94. https://doi.org/10.1093/nar/gki390
50. Nishigaya Y, Fujimoto Z, Yamazaki T. Optimized inhibition assays reveal different inhibitory responses of hydroxylamine oxidoreductases from beta- and gamma-proteobacterial ammonium-oxidizing bacteria. *Biochem Biophys Res Commun*. 2016;**476**(3):127–33. https://doi.org/10.1016/j.bbrc.2016.05.041
51. Sayers EW, Bolton EE, Brister JR, Canese K, Chan J, Comeau DC, *et al*. Database resources of

the national center for biotechnology information. *Nucleic Acids Res*. 2022;**50**:D20–6. https://doi.org/10.1093/nar/gkab1112
